# A Potential Indicator ARRDC2 Has Feasibility to Evaluate Prognosis and Immune Microenvironment in Ovarian Cancer

**DOI:** 10.3389/fgene.2022.815082

**Published:** 2022-05-18

**Authors:** Mengjun Zhang, Yunduo Liu, Yuan Liu, Siyu Hou, Hao Li, Ying Ma, Can Wang, Xiuwei Chen

**Affiliations:** ^1^ Department of Gynecology, Harbin Medical University Cancer Hospital, Harbin, China; ^2^ Department of Gynecology, Beijing Shijitan Hospital, Capital Medical University, Beijing, China

**Keywords:** arrestin domain containing 2, biomarker, immunity, prognosis, ovarian cancer

## Abstract

**Background:** The abnormal expression of α-arrestin protein family plays a key regulatory role in the occurrence and development of many cancers, including colorectal cancer and cervical cancer, and is inseparable from changes in the tumor immune microenvironment. However, the role of ARRDC2, an important member of this family, in the malignant biological process of ovarian cancer (OC) has not been reported, and its role in the change of the immune microenvironment is also unknown.

**Methods:** In this study, HPA, TCGA, GEO and other databases were used to explore the role of ARRDC2 in the prognosis assessment of ovarian cancer. Then, GO, KEGG analysis and GSEA analysis of the biological processes and cell signaling pathways that ARRDC2 may be involved in activated or inhibited. In addition, the TIMER and TISIDB database were used to conduct in-depth research on the role of ARRDC2 in the change of the immune microenvironment of ovarian cancer. The CMap database explored and screened drugs that may be used for treatment. Through cell transfection, CCK-8, Ki-67 immunofluorescence, wound healing, transwell and clone formation assay, the effect of ARRDC2 knockdown on the malignant biological behavior of OC cells were explored.

**Results:** There were significant differences between OC and ARRDC2 mRNA and protein levels. High ARRDC2 expression level is associated with poor overall survival and can be used as an independent prognostic factor. Interestingly, ARRDC2 expression is positively correlated with B cells, Neutrophils, Dendritic cells and CD8+ T cells, signifying that ARRDC2 may be related to infiltration of immune cells. ARRDC2 and its co-expressed genes are enriched in cell signaling pathways related to the immune system. We explored two possible drugs for the treatment of ovarian cancer. Finally, the results of *in vitro* experiments indicated that knockdown of ARRDC2 may inhibit malignant phenotypes such as proliferation and migration of OC cells.

**Conclusion:** The differentially expressed ARRDC2 may be a potential prognostic indicator and can be used as a novel biomarker for exploring the immune microenvironment of ovarian cancer.

## Impact Statement

Ovarian cancer is one of the most common malignant tumors in female reproductive system. Recently, ARRDC gene family is shown in many studies to play a critical role in tumor growth and invasion, which may shed light on study on OV. ARRDC2 is a member of the ARRDC gene family and has been suggested to be involved in various cellular processes of tumorigenesis and progression, such as invasion, migration, proliferation, transformation, and survival. However, little is known about the significance of ARRDC2 in ovarian cancer. Our research may provide new ideas and directions for the diagnosis and treatment of ovarian cancer. ARRDC2 may provide a new development direction for gene targeted therapy and immunotherapy of gynecological tumors. The discovery of a new and effective specific biomarker is of great significance not only for the medical research field of gynecological tumors, but also for the entire medical research field.

## Introduction

In 2018, there were approximately 295,000 new cases of OV (ovarian cancer) and 185,000 deaths in the world. As this tumor is asymptomatic during initial progression and there are no clear early screening methods, it is usually diagnosed at the advanced stage, resulting in an overall 5-years survival rate of less than 40% (Whiteman and Wilson, 2016). The rapid development of high-throughput sequencing technology and transcription research is expected to increase the early diagnosis rate. Although a large number of new proto-oncogenes and tumor suppressor genes that can be used for diagnosis have been discovered, the survival results of ovarian cancer have not been greatly improved. And in terms of treatment, immunotherapy has evolved rapidly over the past 20 years, giving patients with ovarian cancer, known as “immunogenic tumors,” more access to treatment (Santoiemma et al., 2016). However, the response rate of ovarian cancer patients to existing immunotherapy is not satisfactory. Obviously, the understanding of the tumor immune microenvironment of ovarian cancer is still insufficient, and more in-depth research on it and finding specific genes that potentially affect the tumor immune microenvironment and can be used as immunotherapy targets can help improve this situation. In short, it is of great significance to find biomarkers that may be used in early diagnosis and immunotherapy and try to explore their mechanisms.

The mammalian α-arrestin family consists of five structural domain-containing arrestin proteins (ARRDC1-5) and TXNIP. Arrestin domain containing 2 (ARRDC2) is an enigmatic member of the arrestin protein family that plays an important role in the regulation of G protein-coupled receptors (GPCRs) (Dores et al., 2015; Tian et al., 2016). Numerous recent studies have established a link between α-arrestin family and cancer. ARRDC3 and TXNIP were considered to be tumor suppressor genes that regulate a variety of cellular processes. For instance, ARRDC3 was decreased in prostate cancer, breast cancer and colorectal cancer (Huang et al., 2012; Rafiq et al., 2013). However, tumor relevance studies of ARRDC2 have not been reported. Given that members of the arrestin protein family play a momentous role in the biology of tumors, the function of ARRDC2 in tumors, especially in ovarian cancer, has attracted great interest to us.

We are the first study to investigate the important role of the ARRDC2 of the arrestin protein family in the occurrence, development and poor prognosis of ovarian cancer. Here, we performed a deep dive into the TCGA database and the GEO database to determine the impact of ARRDC2 on the progression and poor prognosis of ovarian cancer. The interrelationships between ARRDC2 and immune cell infiltration, immune checkpoints and chemokines were also explored by the Timer database and TISIDBD database. Subsequently, these data were correlated with the clinical regression and prognosis of OV patients. The results showed that high expression in OV and affected the clinical prognosis of patients. Excitingly, a close correlation was found between ARRDC2 expression and the tumor immune microenvironment of the tumor including infiltration of immune cells, immune checkpoint and chemokines. We sought to explore the cellular signaling pathways associated with ARRDC2 and potential small molecule drugs. Finally, the effect of ARRDC2 on the malignant phenotype of OC was confirmed by knockdown of ARRDC2 in OC cell lines. Overall, the results of multiple high-throughput data and a series of rigorous *in vitro* experiments confirmed that ARRDC2 might drive the malignant biological behavior of OC cells. In conclusion, this study attempts to explore potential as a new immune-related prognostic biomarker for OV patients, which may open up a new approach for the combination of immunotherapy and gene therapy for OV patients.

## Materials and Methods

### Data Collection

The Gene Expression Omnibus database (GEO, https://www.ncbi.nlm.nih.gov/geo/) is a world-recognized data-rich public platform, and the public sequencing data in this database have contributed significantly to oncology research. After searching and screening, three data sets (GSE29450, GSE10971, and GSE19829) containing gene expression data were selected. Among them, GSE19829 additionally includes prognostic information such as overall survival time. Microarray data from the GSE29450 (OV = 10, Normal = 10) and GSE10971 (OV = 13, Normal = 24) datasets from GEO were used to study ARRDC2 gene in OV and in normal control. The GSE19829 (OV = 28) data set containing survival information was used in a survival meta-analysis related to the expression level of ARRDC2 by combining with the survival information of TCGA (OV = 372). The Cancer Genome Atlas (TCGA, https://portal.gdc.cancer.gov/) database has a large amount of transcriptomic data such as gene expression data and DNA methylation data. Such a powerful database of massive information has largely improved molecular research of tumor. Therefore, transcriptome data, methylation data and corresponding clinical data of 372 OV patients were collected from the TCGA database. These data were then used to explore the expression level of ARRDC2 and its relationship with specific clinical features and prognosis. At the same time, the relationship between ARRDC2 and changes in methylation sites was also explored.

### Cell Culture and Cell Transfection

Ovarian cancer cell lines (SKOV3 and A2780) and corresponding normal cell lines (IOSE80) were provided by Shanghai Sun Ran HAKATA Cell Bank (http://www.xrshbio.com/). The cells were cultured at 37°C in a 5% CO_2_ incubator using DMEM medium containing 10% fetal bovine serum (FBS. Gibco), 100 U/mL penicillin and 0.1 g/L streptomycin in DMEM medium. When the cells proliferated to about 80%–90% of the bottom of the culture vessel, the cells were passaged and isolated by digestion with 0.25% trypsin. Cells were seeded in 6-well medium plates and 100 pmol siRNA was transfected into each plate using siRNA-Mate (GenePharma, Shanghai,China) following the product instructions standard procedure, and detected the transfection efficiency by real-time quantitative polymerase chain reaction (RT-qPCR) at 24 h after transfection and Western blot at 48 h after transfection. GAPDH was set as an internal reference and its primer sequences were as follows: (GAPDH-F:5′-CAAGGTCATCCATGACAACTTTG-3′, GAPDH-R:5′-GTCCACCACCCTGTTGCTGTAG-3′). The primer sequences of ARRDC2 were as follows: (ARRDC2-516-F:5′-GUGUCCGCUACUGUAUCAATT-3′, ARRDC2-516-R:3′-UUGAUACAGUAGCGGACACTT-5′, ARRDC2-149-F:5′-GACAAGGGUGAAAGCGUUCUTT-3′, ARRDC2-149-R:3 ′-AGA​ACG​CUU​UCA​CCU​UGU​CTT-5′). An empty sequence was constructed as a control (si-NC). 24 h after transfection, the complete medium was replaced according to the instructions, and the knockdown efficiency experiment verified that the ARRDC2-516 sequence was effective and was used for subsequent experiments.

### RT-qPCR

Expression of ARRDC2 in human ovarian cancer cells was detected using RT-qPCR. Total RNA was extracted from the cells using Total RNA Kit I kit (Omega Biotek). RNA reverse transcription was performed under the guidance of NovoScript Plus All-in-one 1st Strand cDNA Synthesis SuperMix (Novoprotein) was performed. The relative expression levels of ARRDC2 were determined by RT-qPCR using NovoStart SYBR qPCR SuperMix Plus (Novoprotein) kit, and GAPDH was used as an internal reference control for ARRDC2 using the 2^−ΔΔCt^ method. The primer sequences of GAPDH and ARRDC2 were as follows: (GAPDH-F: 5′-CAA​GGT​CAT​CCA​TGA​CAA​CTT​TG-3′, GAPDH-R: 5′-GTC​CAC​CAC​CCT​GTT​GCT​GTA​G-3′, ARRDC2-F: 5′- CCC​GAT​CCT​GGT​ACT​GTA​ACC-3′, ARRDC2-R: 5′- CGT​TGT​CGA​TCT​CGG​CAA​AGA-3′). The thermal cycling conditions were as follows: Initial denaturation at 95°C for 10 min, denaturation at 95°C for 10 s, annealing and extension at 60°C for 30 s, for a total of 40 cycles.

### Survival Meta-Analysis

A systematic search in large authoritative databases (such as PubMed and Web of Science) did not reveal any previous studies on the carcinogenicity and poor prognosis of ARRDC2. Therefore, this study combined data from two datasets (GSE19829 and TCGA RNA sequences) in a survival meta-analysis to reveal the prognostic significance of ARRDC2 on OV for the first time. The heterogeneity between studies was assessed by Q test (I^2^ statistics). The fixed effects model is applicable when there is no heterogeneity or I^2^ < 50%. Otherwise, a random-effects model was applied. The random effects model was applied to this study according to the specific situation.

### ARRDC2 Related Gene Enrichment Analysis

Go (gene ontology) and KEGG (Kyoto Encyclopedia of Genes and Genomes) pathway enrichment analysis is carried out using David’s online tool. In short, the list of genes that are positively and negatively related to ARRDC2 obtained through Pearson analysis based on TCGA data was uploaded to the DAVID database for Go and KEGG pathway enrichment analysis. In addition, we applied the “cluster Profiler” R package for GO enrichment analysis. We used Spearman correlation analysis to describe the correlation between quantitative variables without normal distribution. *p*-values less than 0.05 were considered statistically significant. Gene Set Enrichment Analysis (GSEA) is an analytical tool for analyzing cellular signaling pathways developed jointly by MIT and Harvard University. The RNA sequencing data from TCGA is batch-corrected and normalized, and then divided into “H group” (ARRDC2 high expression group) or “L group” (ARRDC2 low expression group). Enrichment analysis was performed using GSEA software (version 4.0.3). The number of changes was set to 1,000 and the genomic database was set to Kyoto Encyclopedia of Genes and Genomes (KEGG) cell signaling pathway (*p* < 0.05 was considered as significantly enriched).

### Immune Databases (TIMER and TISIDB)

The Tumor Immunology Estimation Resource (TIMER; https://cistrome.shinyapps.io/timer) is a rich tumor immunology and genetics database available for automated analysis and visualization of data from the TCGA database of 10,897 pan-cancer samples. Firstly, we analyzed the correlation of ARRDC2 expression with the abundance of six immune cell types (neutrophils, CD4+ T cells, B cells, dendritic cells, CD8+ T cells and macrophages) in OV using the TIMER algorithm. Secondly, we also explored the prognostic value of ARRDC2 in OV patients with different immune cell abundance. In addition, we examined the impact of ARRDC2 gene copy number alterations on immune infiltration. Finally, we explored the co-expression relationship of ARRDC2 with common immune checkpoint-encoding genes including PD-1 (PDCD1), PDL1 (CD274) and PDL2 (PDCD1LG2) in tumor purity-corrected state. Then, TISIDB (http://cis.hku.hk/TISIDB/index.php) were used for verification and analysis the relationship between ARRDC2 and immune infiltrating cells and immune checkpoint. To further explore the relationship between ARRDC2 and immune checkpoints, TISIDB database was mined and validated.

### Immune-Related Kaplan-Meier Survival Analysis

The prognostic value of different expression levels of ARRDC2 is analyzed through this database. Using this database, overall survival (OS) of OV patients were analyzed at different immune cell infiltrations. Patient samples were divided into high and low expression groups according to ARRDC2 gene expression levels and evaluated using Kaplan-Meier survival plots (*p* value < 0.05, false positive rate < 0.05). Hazard ratio (HR) had 95% confidence intervals and log-rank *p* values.

### Co-Expression Analysis and Cmap Analysis

Co-expression analysis was performed by Pearson method, and 10 genes positively and negatively correlated with ARRDC2 were obtained based on correlation coefficients and *p*-values for constructing the correlation between genes and genes. Subsequently, the obtained co-expressed genes were used for the screening and prediction of small molecule drugs by the Connectivity Map (CMap, https://portals.broadinstitute.org/cmap/) database constructed by Prof. Lamb et al. Finally, 2D and 3D structural maps and chemical formulas of the drugs were obtained in the PubChem database.

### Western Blotting

Western blot was used to determine the expression level of ARRDC2 and the efficiency of knockdown. Cell lysis was performed using a lysis solution containing 1% protease inhibitor. After complete lysis on ice for 5 min, the cells were centrifuged for 15 min using a 4°C centrifuge at 12,000 g and the supernatant was immediately collected. The corresponding protein concentrations were quantified using the BCA method (Thermo Fisher Scientific, Waltham, United States). Protein electrophoresis separation was performed using 10% SDS-PAGE gels. The proteins in the gels were then transferred to methanol-treated PVDF membranes. 5% skim milk was used to seal the gels for 1 h. ARRDC2 and GAPDH primary antibodies were mixed with PVDF membranes and incubated overnight in a refrigerator at 4°C. PVDF membranes were washed three times and mixed with horseradish peroxidase-conjugated rabbit secondary antibodies and incubated at room temperature for 1 h. After that, the proteins were washed again with TBST. After that, the membranes were washed again 3 times with TBST. Finally, the membrane was developed with ECL developer and imaged using a charge-coupled camera LAS4000 (Fujifilm, Tokyo, Japan). The grayscale values of the strips were measured by ImageJ software (version v.1.52).

### CCK8 Assay

The cell proliferation ability after transfection was studied by CCK8 (Cell Counting Kit-8). The cell suspension was evenly plated in a 96-well plate in a volume of 100 μl per well. After the cells adhered, 10 μl of CCK-8 reagent (Yeasen, Shanghai, China) was added to the culture medium, and the microplate reader was performed after incubation for 2 h. Set the detection time as 0, 12, 24, and 48 h respectively. OD (optical density) was measured at a wavelength of 450 nm using a microplate reader (Thermo Fisher Scientific, Waltham, United States).

### Clone Formation Assay

Plate the transfected cell suspension evenly in a 6-well plate at 200 cells/well. Cells in 6-well plates were then grown in complete medium. On the 4th day, the medium could be replaced with fresh medium, and cell colony formation was observed after 10 days. Fix cell colonies with 4% paraformaldehyde for 30 min. Cell colonies were stained with 1% crystal violet for 5 min. Formed cell colonies were observed and imaged under a microscope. The number of colonies formed was measured and counted by ImageJ (version 1.52).

### Ki-67 Cell Immunofluorescence

Cells were added evenly in 24-well plates, and when the cell density reached 50–60%, 4% paraformaldehyde was used to fix the cells. Cell permeabilization was performed with 0.5% Triton X-100 for 20 min. After washing with PBS, block with 10% goat serum for 1.5 h at 37°C. Primary antibody (Ki-67, 1:200) was added to each well and incubated overnight at 4°C in the dark. Add secondary antibody and incubate at 37°C for 1 h in the dark. Finally, nuclei were stained with DAPI and incubated in the dark for 10 min. The images were observed under a fluorescence microscope and randomly collected from five fields of view. The numbers of viable and proliferating cells were measured and recorded using ImageJ software (version 1.52).

### Wound-Healing Assay

Transfected ovarian cancer cells were seeded in 6-well plates at the appropriate density. A 200 μl sterile pipette tip was used to make a straight scratch on each well, followed by three washes with 1 × PBS to remove detached cells. Change complete medium to fresh serum-free medium. Scratches at the same site were photographed with a microscope at 0 and 24 h, respectively. Statistical analysis of wound healing rates was performed using ImageJ (version 1.52).

### Transwell Assay

100 μl of the transfected ovarian cancer cell suspension was seeded into the upper chamber of a transwell plate (Corning Costar, Shanghai, China). Add 600 µl of minimal essential medium and 20% FBS to the lower chamber of the transwell plate. Transwell plates were incubated for 24 h at 37°C and 5% CO_2_. Cells that crossed the membrane into the lower compartment were then fixed in 95% ethanol for 15 min. Stain with 0.1% crystal violet for 15 min. Then use a cotton swab to wipe the upper chamber without passing through the cells. Use a microscope to observe the cells and take pictures. Measure and record the number of migrating cells using ImageJ (version 1.52).

### Statistical Analysis

Statistical data analysis was performed using R software (version 3.6.1). Survival and clinicopathological characteristics data were obtained from the TCGA database and the GEO database. Then, the overall survival of ARRDC2 was determined by Kaplan-Meier method. Univariate COX and multivariate COX analyses were used to analyze the factors affecting the prognosis of patients with OV. Student’s *t*-test, Kruskal Wallis test, and Wilcoxon signed-rank test was widely used to compare statistical indicators (*p* < 0.05 was considered to be statistically significant).

## Results

### Correlation Between ARRDC2 Expression and Clinical Characteristics of OV

**FIGURE 1:**
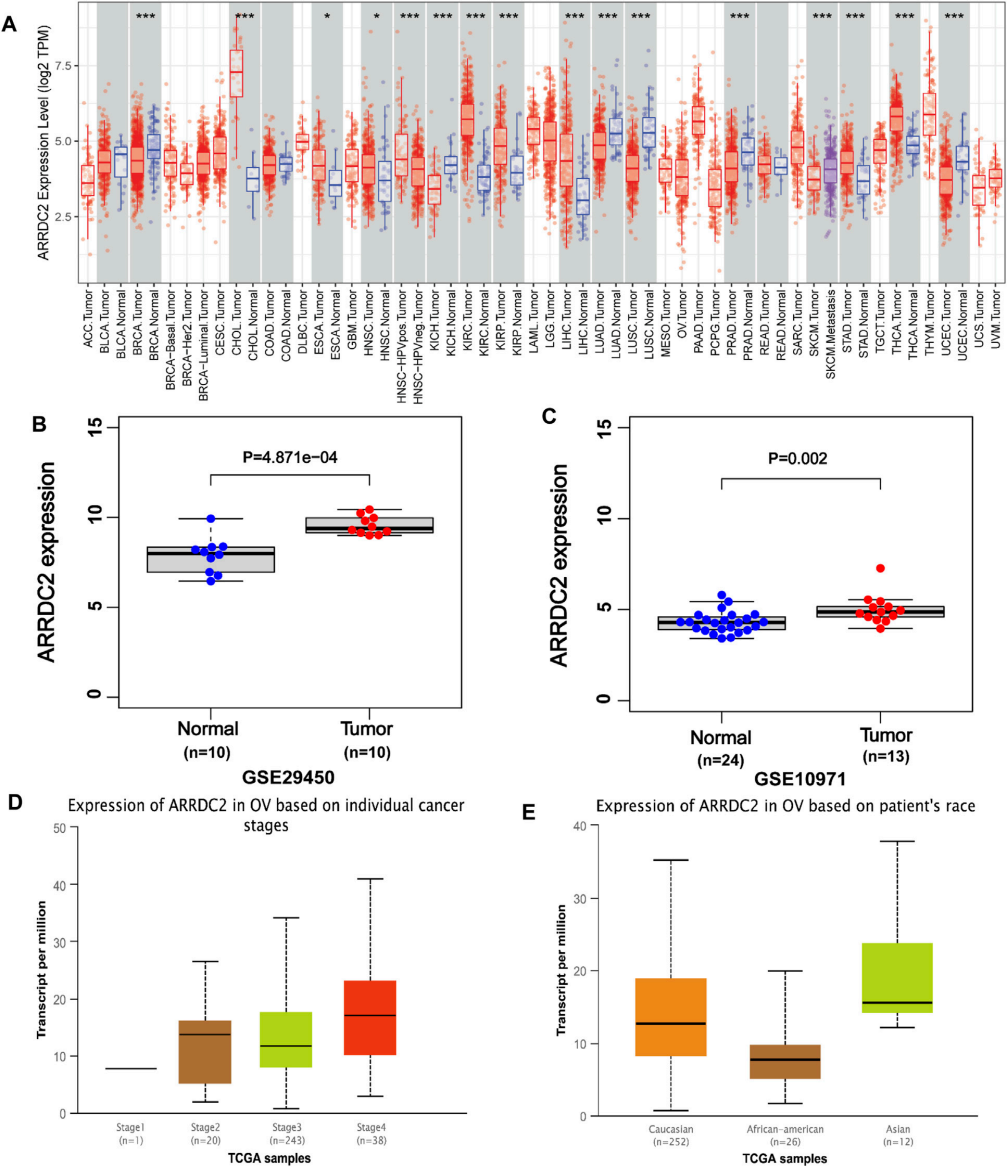
The expression of ARRDC2 (mRNA, gene microarray and gene sequencing) in OVs. **(A)** Expression of ARRDC2 in pan-cancer. **(B)** Box plot based on the expression level of ARRDC2 in the GSE29450 (OV = 10, Normal = 10). **(C)** Box plot based on the expression level of ARRDC2 in the GSE10971 (OV = 13, Normal = 24). **(D)** The expression level of ARRDC2 in OV based on individual FIGO stage. **(E)** The expression level of ARRDC2 in OV based on the race of patient.

ARRDC2 has abnormally high expression in a variety of human malignant tumors (Figure 1A). The analysis of a total of 34 normal ovarian tissues and 23 ovarian cancer tissues from two GEO datasets (GSE29450 and GSE10971), revealed that the ARRDC2 expression was high and statistically significant in tumor tissues (Figures 1B,C). RT-qPCR was performed to verify the results of the above analysis. In addition, the relationship between ARRDC2 expression and clinical characteristics in 361 tumor samples from TCGA in the UALCAN database was explored. Correlation analysis showed that the expression of ARRDC2 was mainly positively correlated with FIGO stage and race (Figures 1D,E). It can be seen that ARRDC2 expression was higher in patients with advanced FIGO stages (III and IV) than early FIGO stages (I and II), detailed clinical features are shown in Supplementary Table S1. In addition, we also explored the differences in ARRDC2 mRNA levels expression between different race groups, and ARRDC2 expression was significantly higher in Asian race than in African American race. In conclusion, our study explored up that ARRDC2 was extremely high expressed in ovarian cancer tumor in TCGA and GEO databases and was closely related to important clinical factors such as FIGO stage. Therefore, further studies on ARRDC2 are needed to explore its value in OV.

### The Prognostic Value of ARRDC2 in OV Patients

**FIGURE 2:**
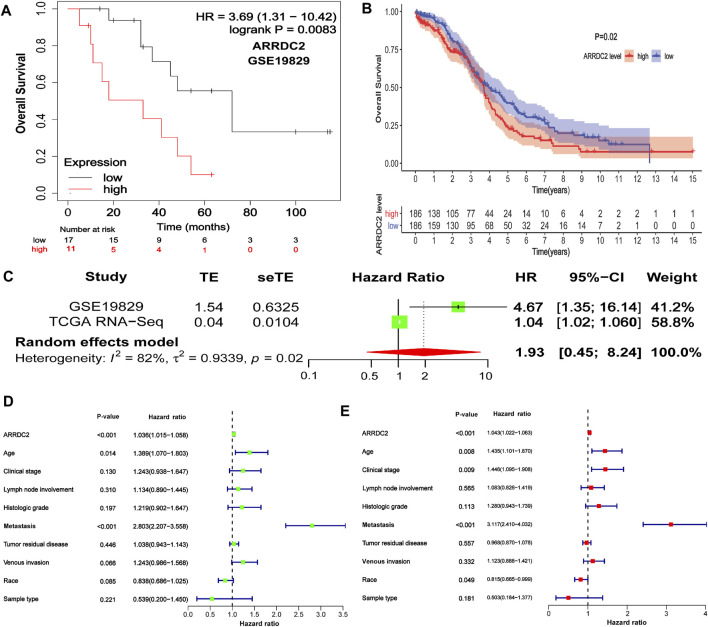
The correlation between ARRDC2 and the poor prognosis of patients with OV. **(A)** The Kaplan-Meier survival curve revealed that the high expression of ARRDC2 lead to a poor prognosis in OVs (GSE19829, *N* = 28, *p* = 0.0083). **(B)** The Kaplan-Meier survival curve revealed that the high expression of ARRDC2 lead to a poor prognosis in OVs (TCGA, *N* = 372, *p* = 0.02). **(C)** Forest plot of high ARRDC2 expression with poor OS in OV patients based on survival meta-analysis of two datasets (GSE19829 and TCGA RNA-Seq, *p* = 0.02). **(D,E)** Analysis of univariate and multivariate factors affecting the prognosis of patients with OV. **(D)** Univariate cox analysis. **(E)** Multivariate cox analysis.

To further explore the prognostic value of ARRDC2 for patients, Kaplan-Meier survival analysis was used to assess the relationship between ARRDC2 expression levels and OS. The KM survival analysis of 372 samples from TCGA showed that high ARRDC2 expression was associated with shorter overall survival in OV patients, *p* = 0.02 (Figure 2B). The GEO data also obtained the same results (Figure 2A). Univariate Cox analysis showed that the high expression of ARRDC2 (HR = 1.036; 95% CI = 1.015–1.058; *p* < 0.001), age (HR = 1.389; 95% CI = 1.070–1.803; *p* = 0.014) and metastasis (HR = 2.803; 95% CI = 2.207–3.558; *p* < 0.001) were high risk factors (Figure 2D). Multivariate Cox analysis showed that the high expression of ARRDC2 (HR = 1.043; 95% CI = 1.022–1.063; *p* < 0.001), age (HR = 1.435; 95% CI = 1.101–1.870; *p* = 0.008) and person neoplasm cancer status (HR = 3.117; 95% CI = 2.410–4.032; *p* < 0.001) were high risk factors (Figure 2E). Overall, it is not difficult to see that ARRDC2 as an independent prognostic risk factor is abnormally high in OV patients.

### Survival Meta-Analysis

Although we have explored the impact of ARRDC2 on the survival outcome of OV patients, to increase the credibility and scientific validity of this study, we collected different mRNA expression data (GSE19829 and TCGA RNA-Seq) for meta-analysis from two datasets, which contained a total of 400 samples. The results showed that high expression of ARRDC2 was a risk factor in patients with OV (HR = 1.93; 95% CI = 0.45–8.24, *p* = 0.02) (Figure 2C). In summary, it can be seen that ARRDC2 can be used as a good biomarker for predicting the overall survival of OV patients.

### Functional Annotation and Signaling Pathway Enrichment Analysis of ARRDC2

**FIGURE 3:**
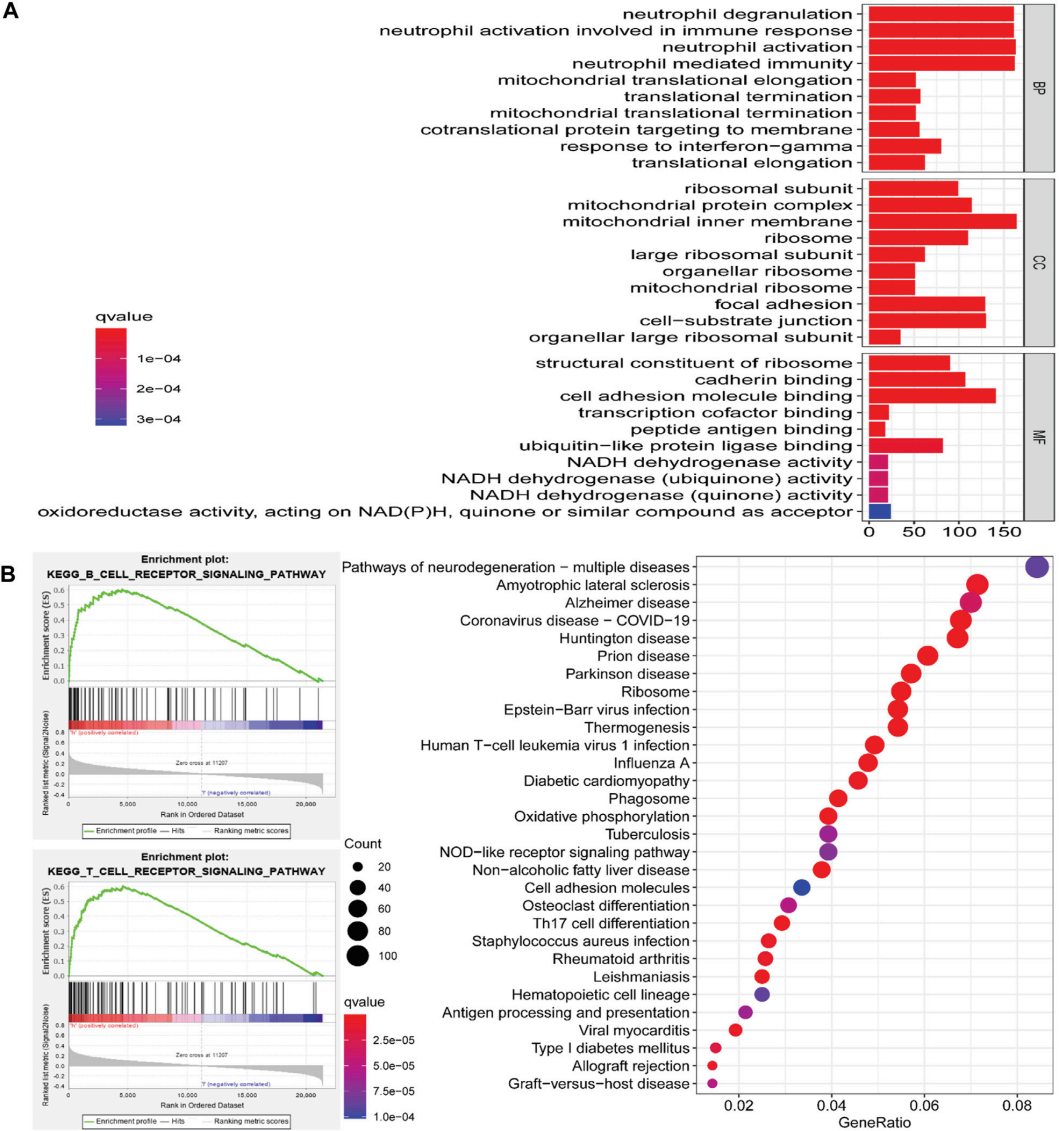
Go (Gene ontology) functional annotation and KEGG (Kyoto Encyclopedia of Genes and Genomes) pathway enrichment analysis of ARRDC2 in OV. **(A)** Go functional annotation. Biological Process, BP (neutrophil degranulation, neutrophil mediated immunity, etc.). Cellular Component, CC (ribosomal subunit, mitochondrial protein complex, ribosome, large ribosomal subunit, etc.). Molecular Function, MF (structural constituent of ribosome, cadherin binding, transcription cofactor binding, etc.). **(B)** KEGG pathway enrichment analysis (B cell receptor signaling pathway, T cell signaling pathway, Human T-cell leukemia virus 1 infection signaling pathway, NOD-Like receptor signaling pathway, Th17 cell differentiation, etc.). GSEA enrichment analysis results of ARRDC2 (B cell receptor signaling pathway and T cell signaling pathway).

To further explore the potential molecular mechanisms of ARRDC2 in tumorigenesis of OV, we attempted to screen a series of pathways and biological functions by co-expressed genes of ARRDC2. The results of Go function annotation analysis are shown in Figure 3A. Among them, those enriched in biological processes included neutrophil degranulation and neutrophil-mediated immunity; those enriched in cellular components included ribosomal subunits, mitochondrial protein complexes, ribosomes and large ribose subunits; those enriched in cellular components included ribosomes, cadherin binding and transcription cofactor binding. The major enriched signaling pathways include B cell, T cell, Human T-cell leukemia virus 1 infection, NOD-Like receptor and Th17 cell differentiation. Importantly, we found that both ARRDC2 enriched functions and signaling pathways are closely related to immunity. In order to further verify the enrichment of ARRDC2 in the immune-related signaling pathways of ovarian cancer, GSEA was used to analyze the data of two groups of OV patients from the TCGA database (ARRDC2 high expression group and ARRDC2 low expression group). The result showed that B cell receptor signaling pathway and T cell signaling pathway were the most important enriched signaling pathways (FDR < 0.25, *p* < 0.05) (Figure 3B). In conclusion, ARRDC2 may affect the malignant progression and poor survival outcomes of patients with ovarian cancer through immunomodulatory effects.

### Relationships of ARRDC2 With Tumor Immune Infiltration

**FIGURE 4:**
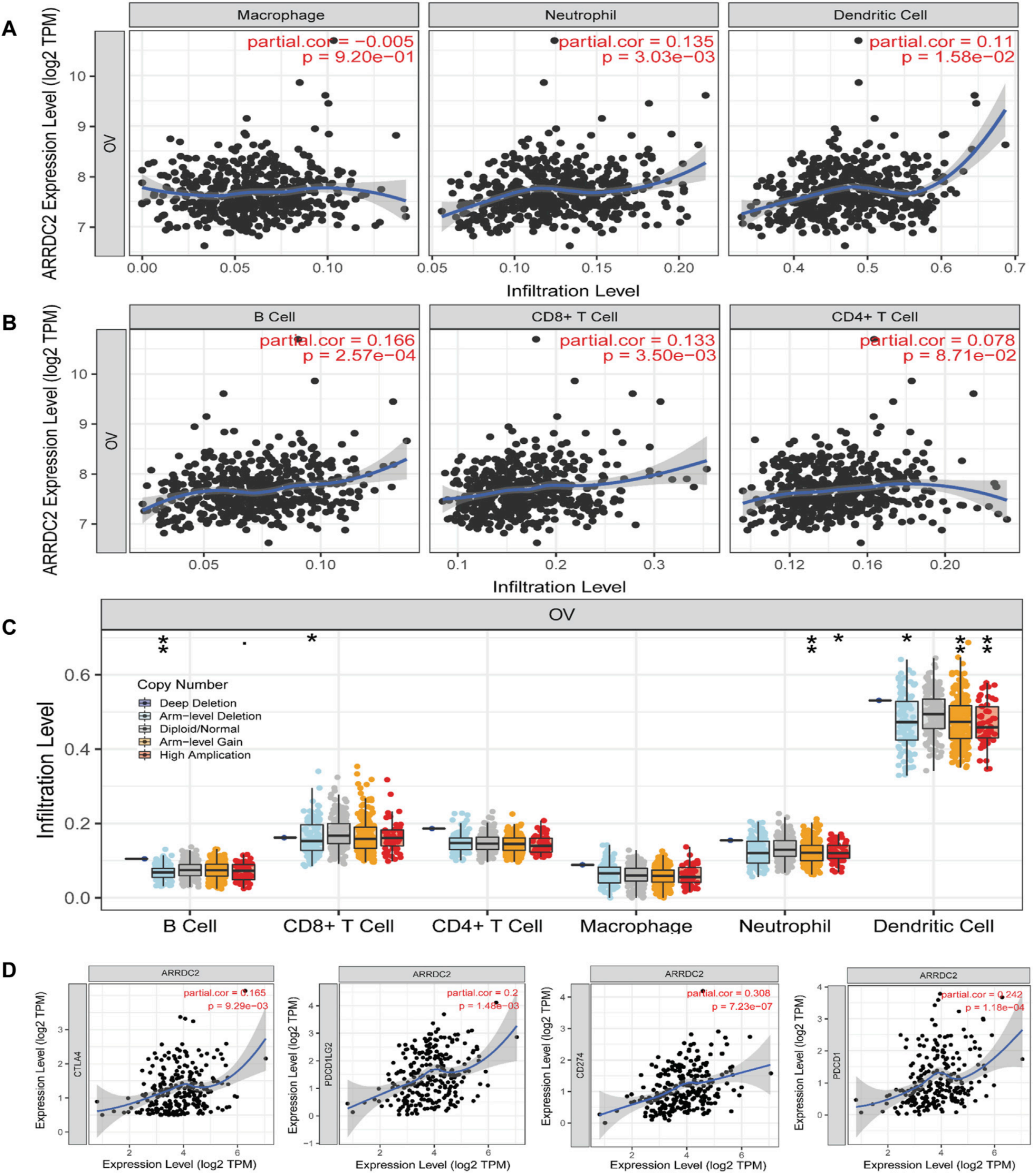
The relationship between the expression of ARRDC2 and proportion of immune infiltrates in TIMER database. **(A,B)** Infiltration of various immune cells. **(A)**Macrophage, Neutrophil, Dendritic Cell. **(B)** B Cell, CD8+ T Cell, CD4+ T Cell. **(C)** Association between ARRDC2 gene copy number and immune cell infiltration levels in OV cohorts (*p < 0.05; **p < 0.01; ***p < 0.001; ****p < 0.0001). **(D)** In TIMER database, the relationship between Immune checkpoint (CTLA4, PDCD1, CD274, PDCD1LG2) and ARRDC2 gene expression was analyzed by Spearman correlation analysis (*p* < 0.05). Correlation coefficients (rho values) and *p* values were shown.

In this study, eight types of infiltrating immune cells in the TIMER database were used to evaluate the relationship between ARRDC2 expression and immunity. The expression level of ARRDC2 was positively related to the infiltration of CD8+ T cells, neutrophils, B cells and dendritic cells (Figures 4A,B). However, there was no significant correlation between ARRDC2 expression and CD4+ T cells and Macrophage. In addition, the SCNA module was chosen to analyze the relationship between the somatic copy number alteration of ARRDC2 and different immune cell infiltrations. As shown in Figure 4C, the somatic copy number alteration of ARRDC2 correlated with infiltration of CD8+ T cells, neutrophils, B cells and dendritic cells. Meanwhile, numerous studies have confirmed that inhibition of the immune checkpoint pathway is an auspicious therapeutic pathway for the induction of effective anti-cancer immunity. Therefore, we analyzed the correlation between the expression levels of ARRDC2 and genes encoding immune checkpoints (Figure 4D), such as PD1 (PDCD1), PDL1 (CD274), PDL2 (PDCD1LG2) and CTLA4. The results showed that the expression levels of ARRDC2 were positively correlated with PD1, PDL1, PDL2, and CTLA4. As mentioned previously, the expression of ARRDC2 was closely correlated with the level of immune infiltration and positively correlated with immune checkpoint.

**FIGURE 5:**
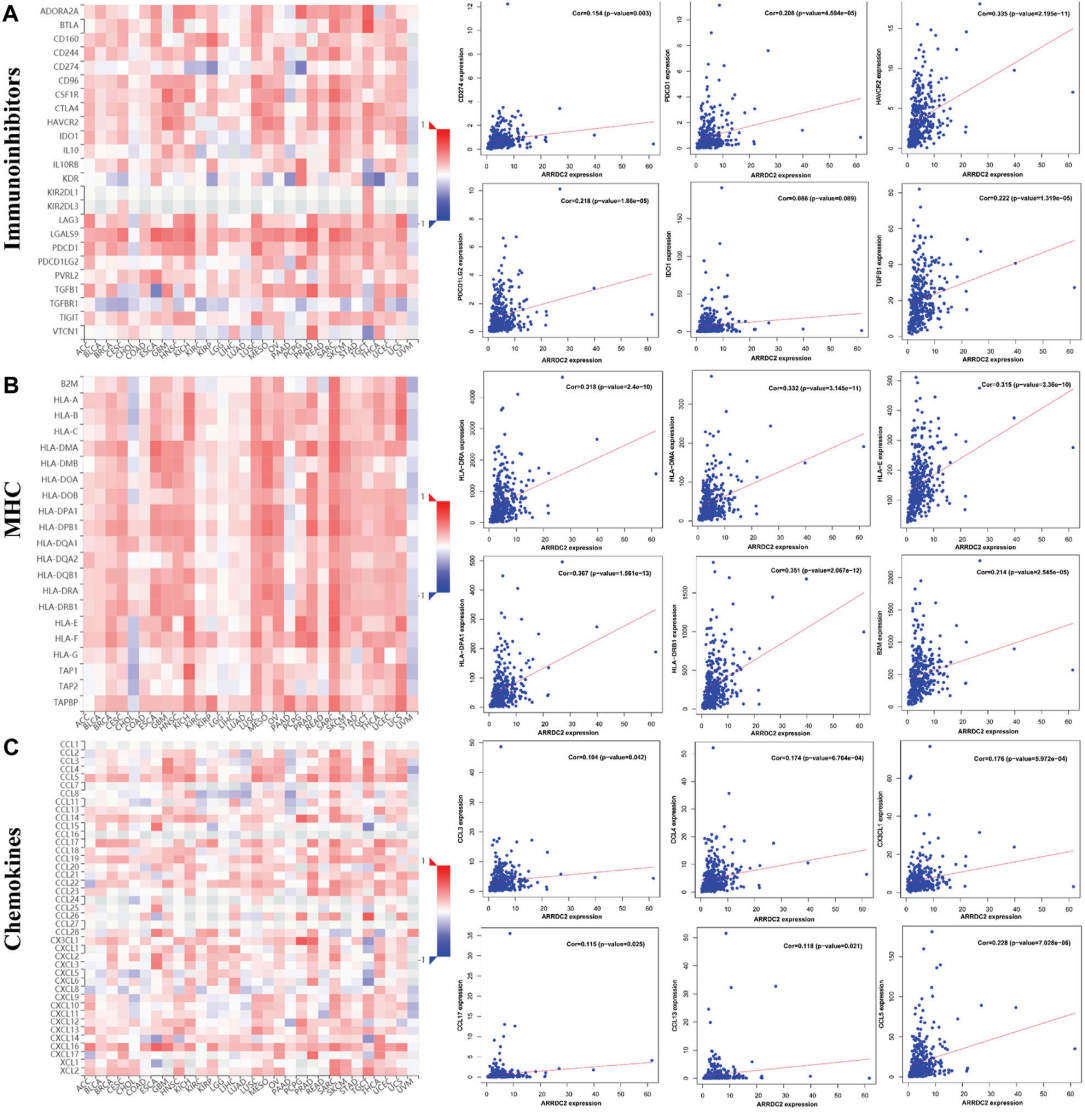
The relationship between the expression of ARRDC2 and immune checkpoints, MHC and chemokines in the TISIDB database. **(A)** The relationship between immune checkpoint (CD274, HAVCR2, IDO1, PDCD1, PDCD1LG2, TGFB1) and ARRDC2 gene expression. **(B)** The relationship between MHC (B2M, HLA-DMA, HLA-DPA1, HLA-DRA, HLA-DRB1, HLA-E) and ARRDC2 gene expression. **(C)** The relationship between Chemokines (CCL17, CCL13, CCL5, CCL3, CCL4, CX3CL1) and ARRDC2 gene expression. Spearman correlation analysis was applied (*p* < 0.05).

**FIGURE 6:**
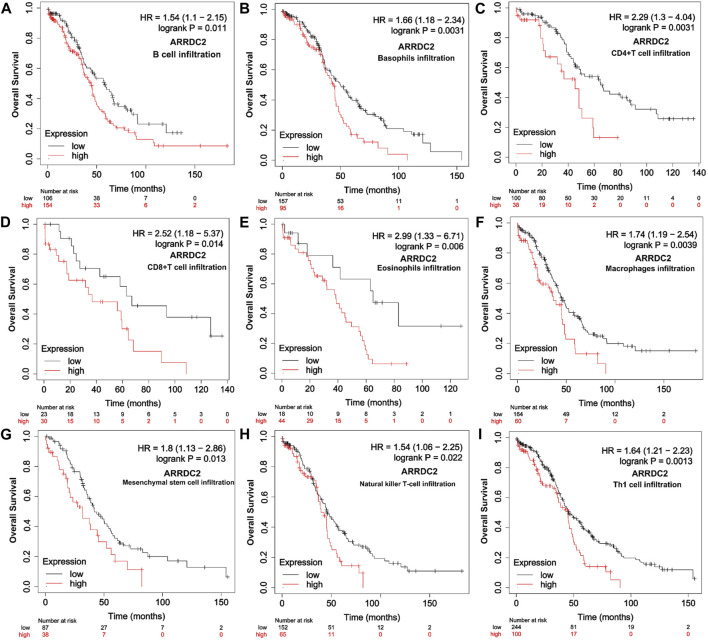
Relationship between overall survival and immune cell infiltration and ARRDC2 gene expression in OV patients. **(A)** B Cell infiltration. **(B)** Basophils infiltration. **(C)** CD4+ T Cell infiltration. **(D)** CD8+ T Cell infiltration. **(E)** Eosinophils infiltration Neutrophil. **(F)** Macrophage infiltration. **(G)** Mesenchymal stem cell infiltration. **(H)** Natural killer T-cell infiltration. **(I)** Th1cell infiltration.

To further verify our speculation, the correlation between ARRDC2 expression and immune infiltration was explored by the TISIDB immune database, and the results were consistent with the TIMER database (Figure 5A). In addition to exploring the relationship between the ARRDC2 gene and immune cell infiltration as well as immune checkpoint using the TISIDB database, we also investigated MHC and chemokines. The results showed (Figures 5B,C) that the ARRDC2 was positively associated with MHC-related genes (B2M, HLA-DMA, HLA-DPA1, HLA-DRA, HLA-DRB1, and HLA-E) and chemokine-related genes (CCL17, CCL13, CCL5, CCL3, CCL4, and CX3CL1). Previous part of this study has shown that ARRDC2 was an independent influence on poor prognosis in OV patients. Thence, we hypothesized that ARRDC2 may affect the prognosis of OV patients partly due to immunological aspects. The Kaplan-Meier plotter database was used to verify the results of the study that the high expression of ARRDC2 during immune cell infiltration leads to a reduction in the overall survival of patients with ovarian cancer. As seen in Figures 6A–I, in the presence of different immune cell infiltration (B Cell, BASOPHILS, CD4+ T Cell, CD8+ T Cell, Eosinophils, Neutrophil, Macrophage, Mesenchymal stem cell, Natural killer T-cell and Th1cell infiltration), patients with high expression of ARRDC2 had shorter OS than those in the low expression group (*p* < 0.05).

### Co-Expression Analysis and Drug Prediction of ARRDC2

**FIGURE 7:**
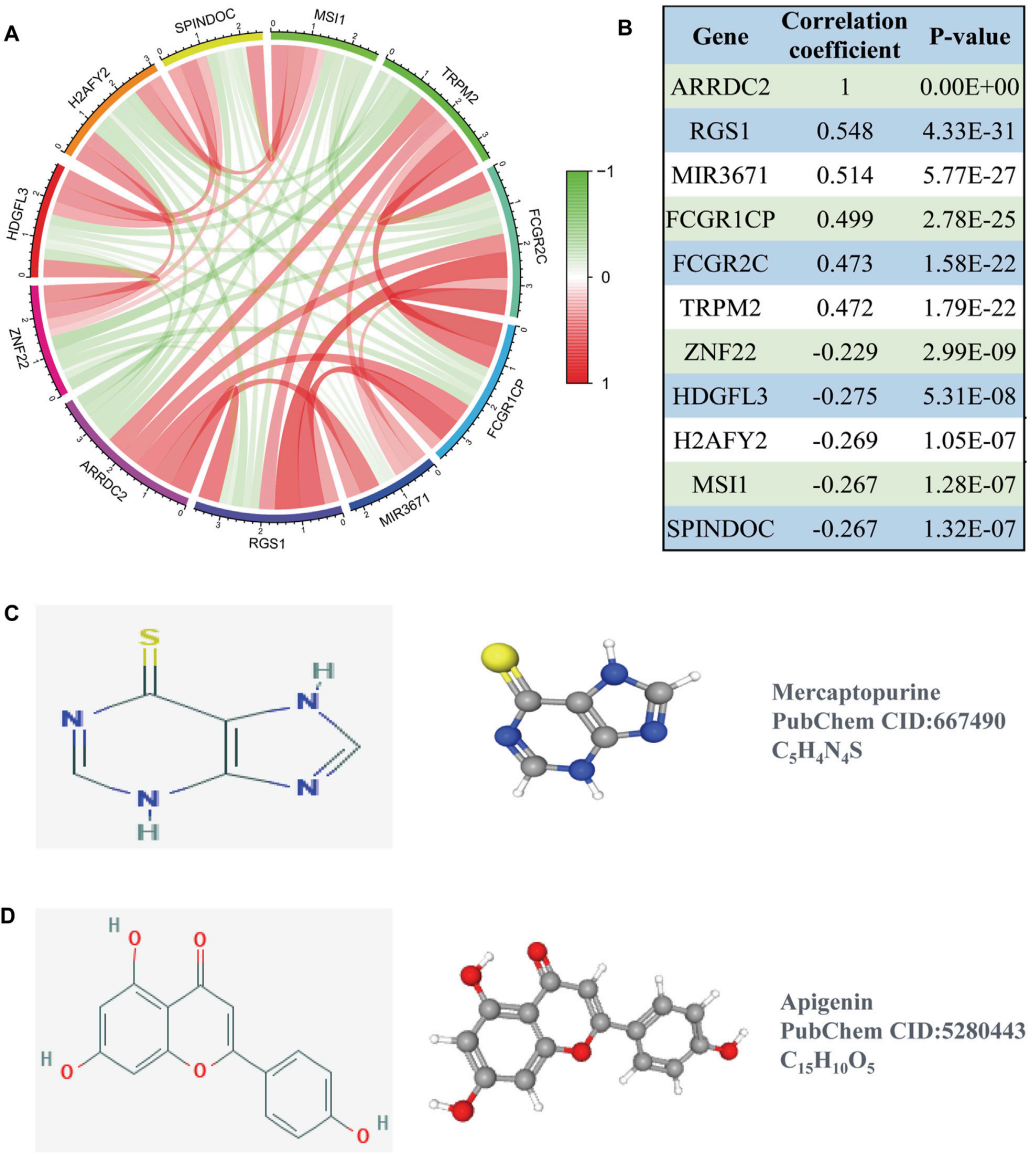
Co-expression analysis of ARRDC2. **(A)** The ten most significant genes of positive and negative correlating with ARRDC2. **(B)** The Correlation coefficients and *p* values of the ten most significant genes of positive and negative correlating with ARRDC2. **(C,D)** Screening of gene therapy drugs for ARRDC2 based on the CMap and Pubchem database (Drug name, chemical structure, 2D structure and 3D structure). **(C)** Mercaptopurine. **(D)** Apigenin.

To better understand the function of ARRDC2, we used co-expression analysis to determine the association of ARRDC2 with other genes. The top five positively and negatively associated genes with ARRDC2 were calculated as shown in the circular plot (Figure 7A). The results showed that ARRDC2 was negatively associated with ZNF22, HDGFL3, H2AFY2, SPINDOC, and MSI1, while positively correlated with TRPM2, FCGR2C, FCGR1CP, MIR3671, and RGS1 (Figure 7B). Subsequently, the co-expressed gene data was used to screen potential gene therapy drugs through the CMap and Pubchem databases. We predicted two possible gene therapy drugs for ARRDC2: Mercaptopurine and Apigenin (Figures 7C,D).

### Expression of ARRDC2 Was Negatively Regulated by DNA Methylation

DNA methylation is one of the most intensively studied epigenetic modifications in mammals, and the importance of altered DNA methylation in tumor formation continues to be revealed. Therefore, we utilized RNA-seq data and DNA methylation data to explore the relationship between DNA CpG site methylation levels and ARRDC2 mRNA expression based on the TCGA data. As shown in Supplementary Figure S2A, ovarian cancer tissues showed low levels of ARRDC2 gene methylation. Two sites were subsequently found to be aberrantly hypermethylated (cg23548920 and cg07374145, Supplementary Figure S2B), the two methylated CpG sites were negatively correlated with ARRDC2 expression (Supplementary Figures S2C,D). In conclusion, DNA methylation of ARRDC2 may be responsible for the difference in ARRDC2 expression levels in normal and tumor tissues.

### Effects of ARRDC2 Knockdown on Malignant Biological Behavior of Ovarian Cancer Cells

**FIGURE 8:**
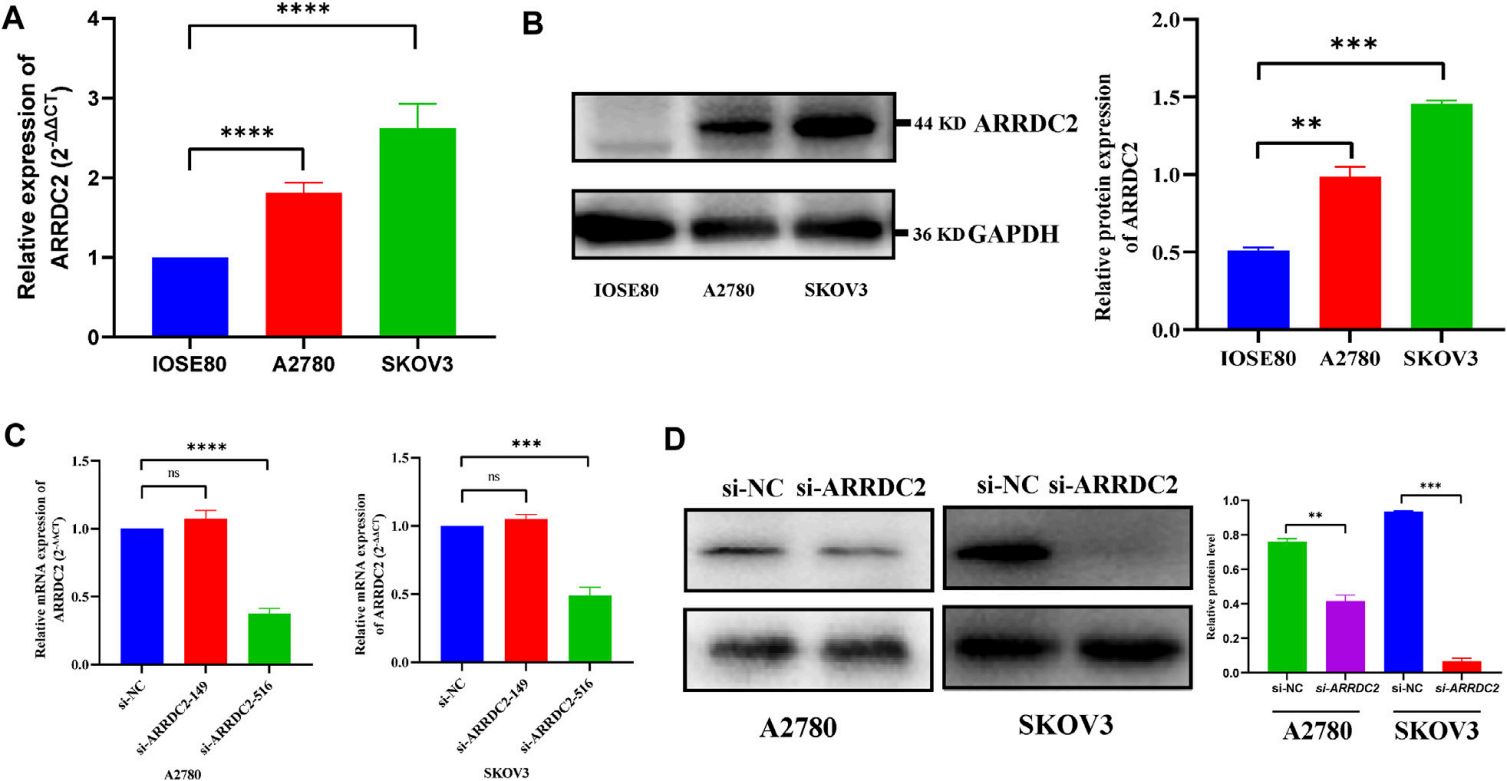
Differential expression and the knockdown efficiency of ARRDC2 at the cellular level. **(A)** RT-qPCR experimental results showed that the expression of ARRDC2 in ovarian cancer cells is higher than that in normal ovarian cells. **(B)** Western blot experimental results showed that the expression of ARRDC2 in ovarian cancer cells is higher than that in normal ovarian cells. The differences in ARRDC2 gene expression levels in OC were compared at the cell levels by Wilcoxon rank sum test. **(C)** RT-qPCR after cell transfection. **(D)** Western blot image and corresponding statistics after cell transfection (*p < 0.05, **p < 0.01, ***p < 0.001, ****p < 0.0001).

**FIGURE 9:**
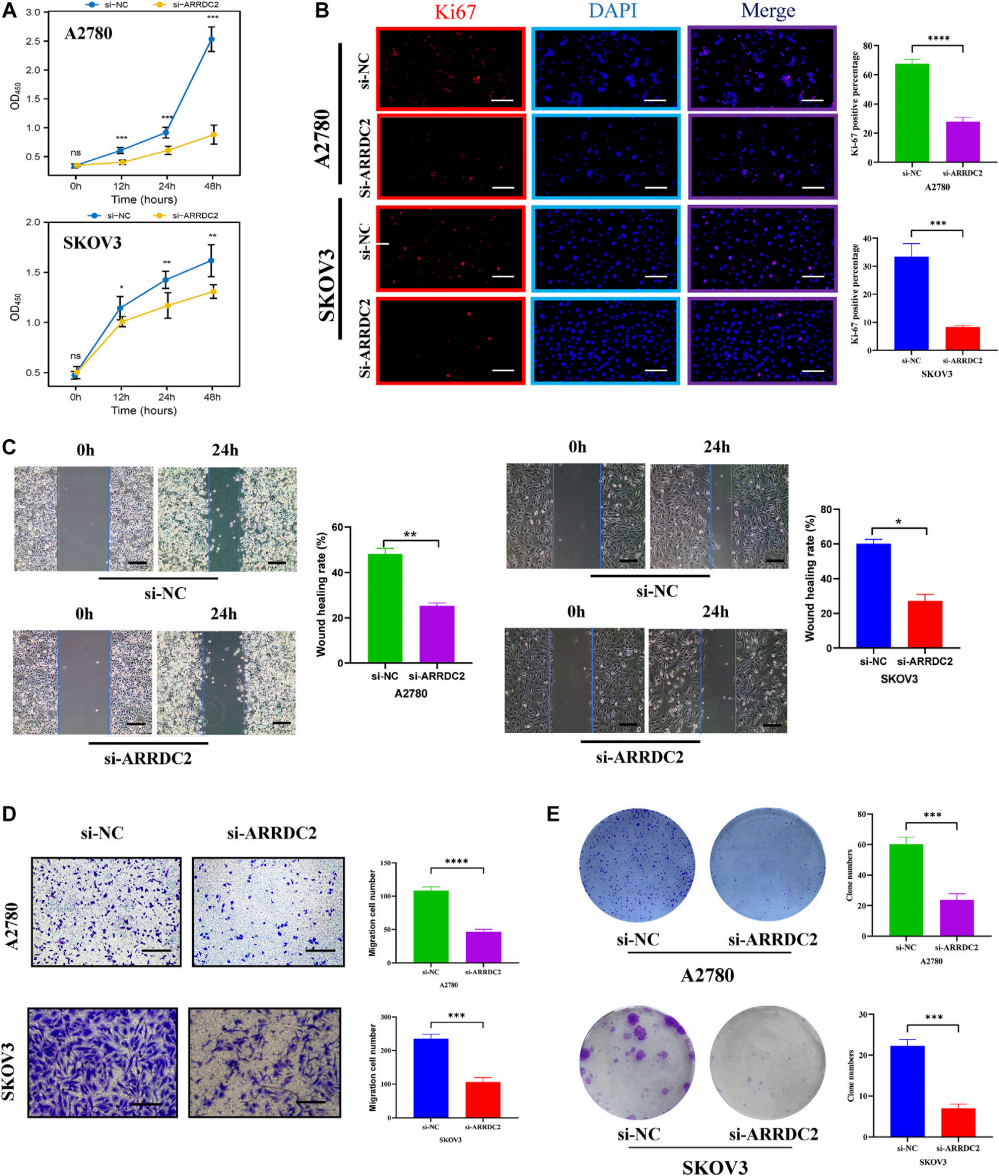
Effects of ARRDC2 gene knockdown on malignant biological behavior of OC cells. **(A)** CCK8 experiment results of A2780 and SKOV3 cell lines after cell transfection. The OD values measured at 450 nm wavelength at 0, 12, 24, and 48 h were displayed, which represented the cell proliferation rate. **(B)** Ki-67 immunofluorescence staining of A2780 and SKOV3 cell lines after cell transfection. **(C)** Wound-healing assay results and statistics of OC cells after cell transfection. The wound-healing rate was measured at 0 and 24 h. **(D)** Transwell assay results and statistics of OC cells after cell transfection. The number of migrating cells was measured at 24 h, which represented the ability to migrate. **(E)** The results and statistics of cell clone formation assay of A2780 and SKOV3 cell lines after cell transfection (*p < 0.05, **p < 0.01, ***p < 0.001, ****p < 0.0001).

On the basis of big data analysis, we used PCR and Western blot experiments to verify that the expression level of ARRDC2 in normal ovarian cells (IOSE80) was significantly lower than that in ovarian cancer cells (A2780 and SKOV3) (Figures 8A,B). After verifying the knockdown efficiency of ARRDC2 (Figures 8C,D), a series of *in vitro* experiments were performed with si-ARRDC2-516 to investigate the effect of ARRDC2 knockdown on the malignant biological behavior of OC cells. First, the CCK8 assay results showed that the optical density of the si-NC group was higher than that of the si-ARRDC2 group at 12, 24, and 48 h in A2780 and SKOV3 cell lines (Figure 9A). Thereafter, the results of colony formation assay and Ki-67 immunofluorescence assay showed that in OC cells, the number of colonies formed (Figure 9E) and the percentage of Ki-67 fluorescence positive cells were higher in the si-NC group compared with the si-ARRDC2 group. high (Figure 9B). Therefore, these results implied that proliferation was inhibited upon ARRDC2 knockdown in OC cells. The wound healing assay results showed that among OC cells, the 24-h wound healing rate of cells in the si-NC group was significantly higher than that in the si-ARRDC2 group (Figure 9C). In addition, transwell assay results showed that in OC cells, the number of migrating cells in the si-NC group was higher than that in the si-ARRDC2 group (Figure 9D). All the above results also confirmed that migration was inhibited after ARRDC2 knockdown in OC cells. Overall, the results of the *in vitro* experiments strongly suggested that biological behaviors such as proliferation and migration were inhibited after ARRDC2 knockdown in OC cells.

## Discussion

The survival outcome of ovarian cancer patients is not promising due to the lack of early screening markers and multiple alternative treatment options. The key role of ARRDC2, an important member of the arrestin protein family related to immunity, in OV needs to be studied urgently. In this study, we tried to use a variety of databases to explore and verify the expression level of ARRDC2, the relationship between ARRDC2 and clinical features and prognosis, potential molecular mechanisms and impact on tumor immune microenvironment. And on the basis of big data analysis, it was verified by *in vitro* cell experiments. The schematic diagram of the flow of this study was shown in Supplementary Figure S1.

The expression level of ARRDC2 in OV was firstly explored. As shown in Figure 1A, ARRDC2 was found to be significantly overexpressed in a variety of malignancies using the TIMER database (Figure 1A). In view of the above pan-cancer results, we tried to verify the expression level of ARRDC2 in OV by other means. Firstly, verify it in the GSE data set related to ovarian cancer. Two GEO datasets (GSE29450 and GSE10970) revealed that ARRDC2 showed significantly higher expression in OV compared to normal controls (Figures 1B,C). In parallel, we performed experimental PCR and Western blot to validate the expression level of ARRDC2 (Figures 8A,B). Secondly, our study also revealed that the abnormally high expression of ARRDC2 may be associated with abnormal hypomethylation of DNA, as shown in Supplementary Figures S2A–D. Numerous studies have confirmed that DNA methylation in epigenetics plays an important role in the malignancy, metastasis and recurrence of ovarian cancer (Papakonstantinou et al., 2020; Reid and Fridley, 2020). Epigenetic regulation, represented by DNA methylation, is essential for the regulation of oncogenes (Oliveira et al., 2021). The above results indicated that ARRDC2 was highly expressed in OV, suggesting that ARRDC2 is a potential oncogene and is affected by methylation.

After discovering that ARRDC2 was a potential oncogene, we tried to explore its impact on the prognosis of OV patients through retrospective studies based on gene expression and clinical information based on TCGA data and GSE19829. The results of the correlation analysis of clinical features showed that the expression level of ARRDC2 in OV increased with increasing FIGO stage and was highly expressed in Asian race groups (Figures 1D,E). And numerous studies have confirmed that the higher the FIGO stage, the worse the prognosis (Gąsowska-Bajger et al., 2021). In view of the above phenomena, it was of interest to us whether ARRDC2 might contribute to the poor prognosis of OV patients. Immediately after, the Kaplan-Meier survival analysis and survival meta-analysis of this study showed that the overall survival of patients in the high-expression group of ARRDC2 was shorter than that of the low-expression group (Figures 2A–C). In addition, survival meta-analysis further improved the scientific validity and rigor of Kaplan-Meier survival analysis. However, to exclude the effect of chance factors, univariate cox and multivariate cox analyses were used to confirm that ARRDC2 could serve as an independent risk factor for poor prognosis in patients with OV (Figures 2D,E). A great many studies have shown that genes in the ARRDC family contributed to the progression of various malignancies such as gastric, cervical and colorectal cancers, and were strongly associated with poor prognosis (Shen et al., 2018; Xiao et al., 2018; Takeuchi et al., 2019). For instance, ARRDC3, a member of the ARRDC family, served as a biomarker for the diagnosis and prognosis of epithelial ovarian cancer (Chen et al., 2021). From this, it can be boldly speculated that ARRDC2 may likewise act as an oncogene in ovarian cancer and lead to poor prognosis, but its possible oncogenic mechanisms need to be further explored.

To further understand the pathological mechanism of poor prognosis of OV due to ARRDC2, we performed GO annotation analysis and enrichment analysis of KEGG cell signaling pathway. As shown in Figures 4A,B, ARRDC2 may be involved in immune response regulatory signaling pathway, B cell receptor signaling pathway, T cell signaling pathway, Th17 cell differentiation and other immune-related biological pathways and processes. Meanwhile, using GSEA, we again confirmed that ARRDC2 was highly and consistently enriched in immune-related signaling pathways. Studies have shown that ARRDC3, an important member of the ARRDC family, was closely related to immunity in epithelial ovarian cancer, which supports our research (Chen et al., 2021). The above enrichment analysis suggested that ARRDC2 may play an oncogenic role in ovarian cancer by influencing immune factors in the tumor microenvironment.

Therefore, we further explored the relevance of ARRDC2 to immune cells and immune checkpoints in the tumor immune microenvironment of ovarian cancer using the TIMER and TISIDB databases (Ru et al., 2019). Figures 4A,B showed that the mRNA expression levels of ARRDC2 positively correlated with the infiltration of immune cells, including CD8+ T cells, B cells, neutrophils and dendritic cells. Numerous studies have confirmed that immune infiltration is considered as one of the hallmarks of cancer. Considering the importance of immune cell infiltration in tumors, TISIDB further evaluated the abundance ratio of different immune cells in ovarian cancer (Figure 5A) (Hanahan and Weinberg, 2011). In addition to this, the TISIDB database also showed a significant positive association of the ARRDC2 gene with immunomodulators, major histocompatibility complex molecules (MHC) and chemokines (Figures 5B,C). Previous studies have shown that if upregulation of the MHC-I complex is present, then NK cells can target these cells to send inhibitory signals, leading to long-term survival of tumor cells (Buller et al., 2020). It also corroborated our study that ARRDC2 was positively correlated with the MHC gene and could act as an oncogene in ovarian cancer. Interestingly, we also found a significant positive correlation between ARRDC2 expression and four immune checkpoints (Figure 4D). With advances in immunotherapy, particularly antibodies against the immune checkpoints cytotoxic, such as T lymphocyte-associated protein 4 (CTLA-4), programmed death protein 1 (PD-1) and programmed death ligand 1 (PD-L1), have shown clinical efficacy in ovarian cancer (Peyraud and Italiano, 2020; Wan et al., 2021). The degree of immune cell infiltration can be determined by gene expression profiles of immune-related genes, which may help estimate the prognosis of patients (Sui et al., 2020). In this regard, this research showed that the overall survival of patients with high ARRDC2 expression was shortened as seen by the Kaplan-Meier Plotter database under different immune cell infiltration scenarios (Figures 6A–I). In conclusion, this study fully confirmed the close correlation between ARRDC2 and important tumor immune microenvironment components such as immune cell infiltration and immune checkpoints. Moreover, the high expression of ARRDC2 could lead to poor prognosis such as reduced overall survival under different immune cell infiltration environments, which suggested that ARRDC2 may be used as a novel immunotherapy target to improve the clinical prognosis of OV patients.

After exploring the aberrant expression of ARRDC2 and its prognostic significance, the effect of ARRDC2 on the biological behavior of OC cells was assessed by a complete set of cytological experiments. The results in Figures 8C,D confirmed that the knockdown efficiency of ARRDC2 was sufficient and provided a basis for further studies. Furthermore, the experiments confirmed the inhibitory effect of ARRDC2 knockdown on the proliferation and migration of OC cells (Figure 9). Through the combination of big data analysis and cell experiment validation, ARRDC2 was preliminarily confirmed as a potential oncogene. May lead to poor prognosis by promoting the malignant biological behavior of OC cells.

The ultimate goal of the study was to benefit the clinic. Therefore, based on the above research part, the CMap small molecule drug analysis was performed using co-expressed genes positively and negatively associated with ARRDC2, and finally two small molecule compounds with potential therapeutic effects on ovarian cancer were predicted: Mercaptopurine and Apigenin (Figure 7). A large number of previous studies have evaluated the reliability of the CMap small molecule drug analysis for drug prediction (Iskar et al., 2010; Cheng et al., 2014). And encouragingly, previous studies have demonstrated the use of Mercaptopurine as an immunomodulatory agent in the treatment of patients with inflammatory bowel disease (Su et al., 2000). In addition, Mercaptopurine is effective in the treatment of immune modulation disorders and acute lymphoblastic leukemia (Marinaki and Arenas-Hernandez, 2020). For Apigenin, it has been reported that Apigenin inhibited various human cancers *in vitro* and *in vivo* as well as stimulates immune responses through multiple biological effects (Yan et al., 2017). Besides, Apigenin limited melanoma growth by inhibiting PD-L1 expression through modulation of tumor and antigen (Xu et al., 2018). Notably, previous studies are highly consistent with our study that ARRDC2 may serve as a new target for immunotherapy and may provide a new direction for subsequent immunopharmacological treatment of ovarian cancer.

The research focus of ovarian cancer treatment is precision immunotherapy or targeted therapy driven by specific biomarkers, which will bring better survival results for patients with ovarian cancer. In addition, it is important to explore new biomarkers not only for prognostic assessment, but also for the exploration of the subtle mechanisms of epigenetic and immunological changes that occur in the tumor immune microenvironment. Based on these circumstances, this study has certain strengths and limitations. The main strength of this study is the originality of the findings. This study brought the role of ARRDC2 as an immune-related prognostic biomarker in ovarian cancer to the public eye for the first time. The discovery and in-depth study of ARRDC2 would enable it to predict the effect of treatment, be used as a new potential therapeutic target and extend the overall survival of patients. However, this study still has some limitations. Although this study clarified the abnormal expression of ARRDC2 and its prognostic significance based on public data and cell experiment data. However, this study did not verify the prognostic significance of ARRDC2 in a clinical cohort. This is the limitation of this study and the direction of further research in the future.

## Conclusion

Our study suggests that aberrantly expressed ARRDC2 may be a potential prognostic marker for OV. More importantly, it may promote the proliferation and migration of ovarian cancer cells and may be associated with the tumor immune microenvironment. Clinically significant ARRDC2 may be used to assess the clinical prognosis of patients with OV and may also be used as a target for immunotherapy or as a potential marker for checkpoint inhibitor-based immunotherapy.

## Data Availability

The datasets presented in this study can be found in online repositories. The names of the repository/repositories and accession number(s) can be found in the article/Supplementary Material.

## References

[B1] Buller C. W. Mathew P. A. Mathew S. O. (2020). Roles of NK Cell Receptors 2B4 (CD244), CS1 (CD319), and LLT1 (CLEC2D) in Cancer. Cancers (Basel) 12, 12. 10.3390/cancers12071755 PMC7409338 32630303

[B2] Chen Y. Tian D. Chen X. Tang Z. Li K. Huang Z. (2021). ARRDC3 as a Diagnostic and Prognostic Biomarker for Epithelial Ovarian Cancer Based on Data Mining. Ijgm Vol. 14, 967–981. 10.2147/ijgm.s302012 PMC7997607 33790626

[B3] Cheng J. Yang L. Kumar V. Agarwal P. (2014). Systematic Evaluation of Connectivity Map for Disease Indications. Genome Med. 6, 540. 10.1186/s13073-014-0095-1 25606058 PMC4278345

[B4] Dores M. R. Lin H. Grimsey F. Trejo J. (2015). The α-arrestin ARRDC3 Mediates ALIX Ubiquitination and G Protein-Coupled Receptor Lysosomal Sorting. MBoC 26, 4660–4673. 10.1091/mbc.e15-05-0284 26490116 PMC4678022

[B5] Gąsowska-Bajger B. Gąsowska-Bodnar A. Knapp P. Bodnar L. (2021). Prognostic Significance of Survivin Expression in Patients with Ovarian Carcinoma: A Meta-Analysis. J. Clin. Med. 10, 10. 10.3390/jcm10040879 PMC7924601 33669912

[B6] Hanahan D. Weinberg R. A. (2011). Hallmarks of Cancer: the Next Generation. Cell 144, 646–674. 10.1016/j.cell.2011.02.013 21376230

[B7] Huang C.-N. Huang S.-P. Pao J.-B. Chang T.-Y. Lan Y.-H. Lu T.-L. (2012). Genetic Polymorphisms in Androgen Receptor-Binding Sites Predict Survival in Prostate Cancer Patients Receiving Androgen-Deprivation Therapy. Ann. Oncol. 23, 707–713. 10.1093/annonc/mdr264 21652578

[B8] Iskar M. Campillos M. Kuhn M. Jensen L. J. van Noort V. Bork P. (2010). Drug-induced Regulation of Target Expression. PLoS Comput. Biol. 6. 10.1371/journal.pcbi.1000925 PMC2936514 20838579

[B9] Marinaki A. M. Arenas-Hernandez M. (2020). Reducing Risk in Thiopurine Therapy. Xenobiotica 50, 101–109. 10.1080/00498254.2019.1688424 31682552

[B10] Oliveira D. Hentze J. O'Rourke C. J. Andersen J. B. Høgdall C. Høgdall E. V. (2021). DNA Methylation in Ovarian Tumors-A Comparison between Fresh Tissue and FFPE Samples. Thousand Oaks, Calif): Reproductive sciences. 10.1007/s43032-021-00589-0 PMC8526488 33891290

[B11] Papakonstantinou E. Androutsopoulos G. Logotheti S. Adonakis G. Maroulis I. Tzelepi V. (2020). DNA Methylation in Epithelial Ovarian Cancer: Current Data and Future Perspectives. Curr. Mol. Pharmacol. 14 (6), 1013–1027. 10.2174/1874467213666200810141858 32778046

[B12] Peyraud F. Italiano A. (2020). Combined PARP Inhibition and Immune Checkpoint Therapy in Solid Tumors. Cancers (Basel) 12. 10.3390/cancers12061502 PMC7352466 32526888

[B13] Rafiq S. Tapper W. Collins A. Khan S. Politopoulos I. Gerty S. (2013). Identification of Inherited Genetic Variations Influencing Prognosis in Early-Onset Breast Cancer. Cancer Res. 73, 1883–1891. 10.1158/0008-5472.can-12-3377 23319801 PMC3601979

[B14] Reid B. M. Fridley B. L. (2020). DNA Methylation in Ovarian Cancer Susceptibility. Cancers (Basel) 13. 10.3390/cancers13010108 PMC7795210 33396385

[B15] Ru B. Wong C. N. Tong Y. Zhong J. Y. Zhong S. S. W. Wu W. C. (2019). TISIDB: an Integrated Repository Portal for Tumor-Immune System Interactions. Bioinforma. Oxf. Engl. 35, 4200–4202. 10.1093/bioinformatics/btz210 30903160

[B16] Santoiemma P. P. Reyes C. Wang L.-P. McLane M. W. Feldman M. D. Tanyi J. L. (2016). Systematic Evaluation of Multiple Immune Markers Reveals Prognostic Factors in Ovarian Cancer. Gynecol. Oncol. 143, 120–127. 10.1016/j.ygyno.2016.07.105 27470997

[B17] Shen X. Sun X. Sun B. Li T. Wu G. Li Y. (2018). ARRDC3 Suppresses Colorectal Cancer Progression through Destabilizing the Oncoprotein YAP. FEBS Lett. 592, 599–609. 10.1002/1873-3468.12986 29364502

[B18] Su C. G. Stein R. B. Lewis J. D. Lichtenstein G. R. (2000). Azathioprine or 6-mercaptopurine for Inflammatory Bowel Disease: Do Risks Outweigh Benefits? Dig. Liver Dis. 32, 518–531. 10.1016/s1590-8658(00)80010-9 11057928

[B19] Sui S. An X. Xu C. Li Z. Hua Y. Huang G. (2020). An Immune Cell Infiltration-Based Immune Score Model Predicts Prognosis and Chemotherapy Effects in Breast Cancer. Theranostics 10, 11938–11949. 10.7150/thno.49451 33204321 PMC7667685

[B20] Takeuchi F. Kukimoto I. Li Z. Li S. Li N. Hu Z. (2019). Genome-wide Association Study of Cervical Cancer Suggests a Role forARRDC3gene in Human Papillomavirus Infection. Hum. Mol. Genet. 28, 341–348. 10.1093/hmg/ddy390 30412241

[B21] Tian X. Irannejad R. Bowman S. L. Du Y. Puthenveedu M. A. von Zastrow M. (2016). The α-Arrestin ARRDC3 Regulates the Endosomal Residence Time and Intracellular Signaling of the β2-Adrenergic Receptor. J. Biol. Chem. 291, 14510–14525. 10.1074/jbc.m116.716589 27226565 PMC4938174

[B22] Wan C. Keany M. P. Dong H. Al-Alem L. F. Pandya U. M. Lazo S. (2021). Enhanced Efficacy of Simultaneous PD-1 and PD-L1 Immune Checkpoint Blockade in High-Grade Serous Ovarian Cancer. Cancer Res. 81, 158–173. 10.1158/0008-5472.CAN-20-1674 33158814 PMC7878408

[B23] Whiteman D. C. Wilson L. F. (2016). The Fractions of Cancer Attributable to Modifiable Factors: A Global Review. Cancer Epidemiol. 44, 203–221. 10.1016/j.canep.2016.06.013 27460784

[B24] Xiao J. Shi Q. Li W. Mu X. Peng J. Li M. (2018). ARRDC1 and ARRDC3 Act as Tumor Suppressors in Renal Cell Carcinoma by Facilitating YAP1 Degradation. Am. J. Cancer Res. 8, 132–143. 29416926 PMC5794727

[B25] Xu L. Zhang Y. Tian K. Chen X. Zhang R. Mu X. (2018). Apigenin Suppresses PD-L1 Expression in Melanoma and Host Dendritic Cells to Elicit Synergistic Therapeutic Effects. J. Exp. Clin. Cancer Res. 37, 261. 10.1186/s13046-018-0929-6 30373602 PMC6206930

[B26] Yan X. Qi M. Li P. Zhan Y. Shao H. (2017). Apigenin in Cancer Therapy: Anti-cancer Effects and Mechanisms of Action. Cell Biosci. 7, 50. 10.1186/s13578-017-0179-x 29034071 PMC5629766

