# Corrigendum: A potential indicator ARRDC2 has feasibility to evaluate prognosis and immune microenvironment in ovarian cancer

**DOI:** 10.3389/fgene.2022.978493

**Published:** 2022-08-24

**Authors:** Mengjun Zhang, Yunduo Liu, Yuan Liu, Siyu Hou, Hao Li, Ying Ma, Can Wang, Xiuwei Chen

**Affiliations:** ^1^ Department of Gynecology, Harbin Medical University Cancer Hospital, Harbin, China; ^2^ Department of Gynecology, Beijing Shijitan Hospital, Capital Medical University, Beijing, China

**Keywords:** arrestin domain containing 2, biomarker, immunity, prognosis, ovarian cancer

In the published article, there was an error in Figure 9 as published. Figure 9C in the published article showed the results of wound healing assays in ovarian cancer cell lines A2780 and SKOV3 after knockdown of ARRDC2. There was an error in Figure 9C in the published article, the image of wound healing in the ovarian cancer cell line SKOV3 at 0 h after knockdown of ARRDC2 was wrong. The corrected Figure 9 and its caption appear below.

**FIGURE 9:**
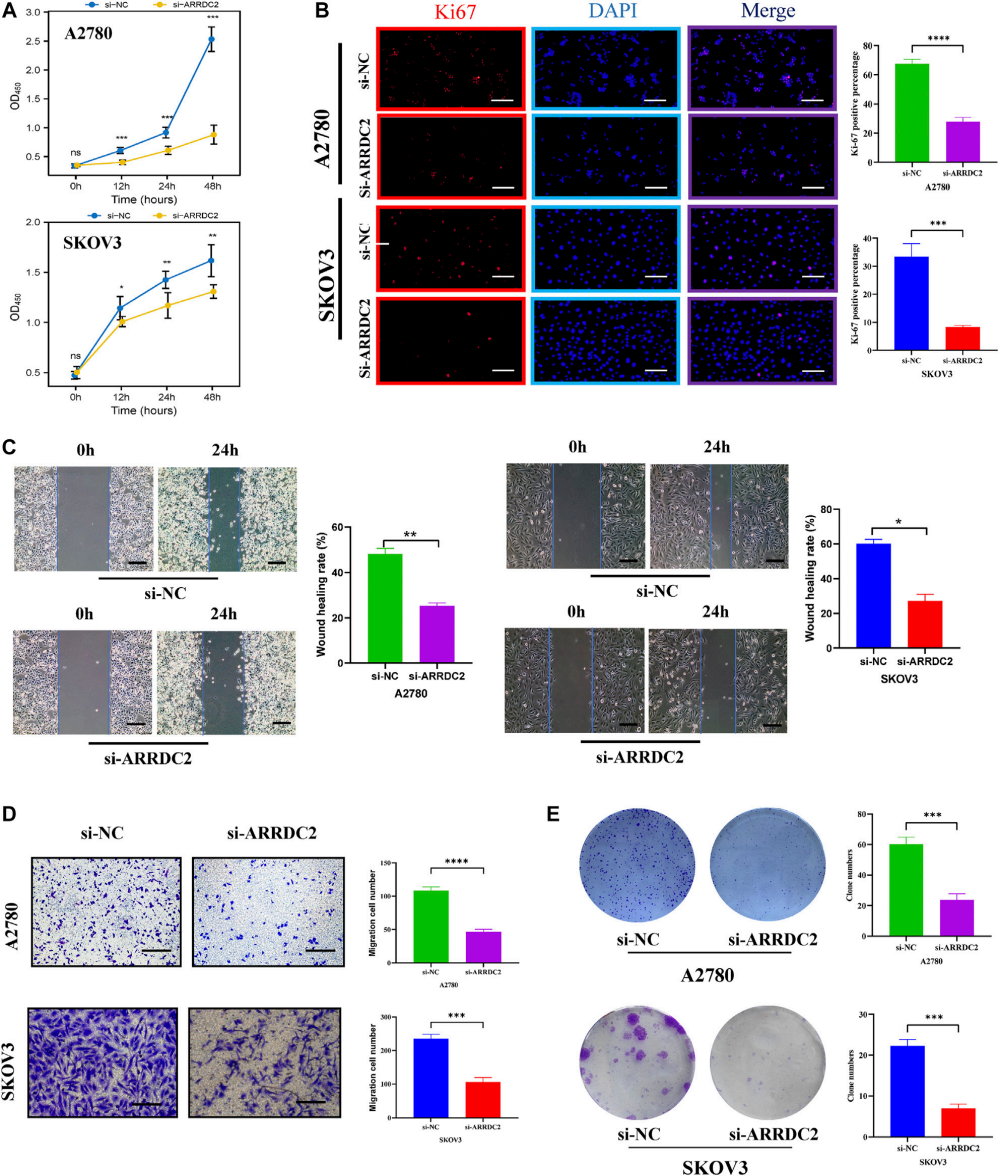
Effects of ARRDC2 gene knockdown on malignant biological behavior of OC cells. **(A)** CCK8 experiment results of A2780 and SKOV3 cell lines after cell transfection. The OD values measured at 450 nm wavelength at 0, 12, 24, and 48 h were displayed, which represented the cell proliferation rate. **(B)** Ki-67 immunofluorescence staining of A2780 and SKOV3 cell lines after cell transfection. **(C)** Wound-healing assay results and statistics of OC cells after cell transfection. The wound-healing rate was measured at 0 and 24 h. **(D)** Transwell assay results and statistics of OC cells after cell transfection. The number of migrating cells was measured at 24 h, which represented the ability to migrate. **(E)** The results and statistics of cell clone formation assay of A2780 and SKOV3 cell lines after cell transfection (*p < 0.05, **p < 0.01, ***p < 0.001, ****p < 0.0001).

The authors apologize for this error and state that this does not change the scientific conclusions of the article in any way. The original article has been updated.

