# Supplementary material for: A Potential Indicator ARRDC2 Has Feasibility to Evaluate Prognosis and Immune Microenvironment in Ovarian Cancer
Source: Front Genet. 2022 May 18;13:815082. doi: 10.3389/fgene.2022.815082 (PMC9157644; doi:10.3389/fgene.2022.815082)

Example of original western blot

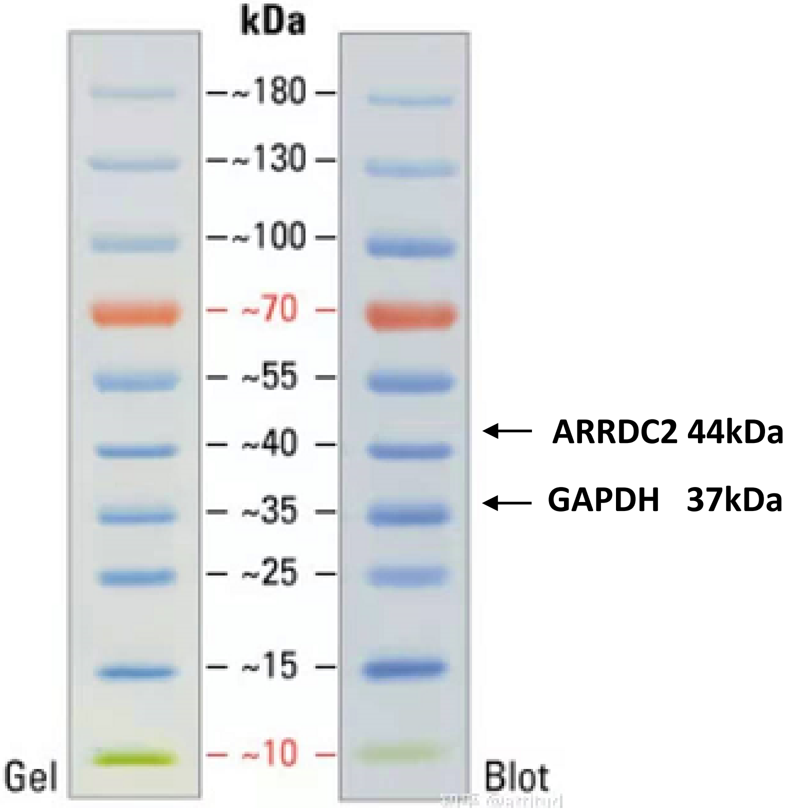

IOSE80 A2780 SKOV3 （Figure 8.B）

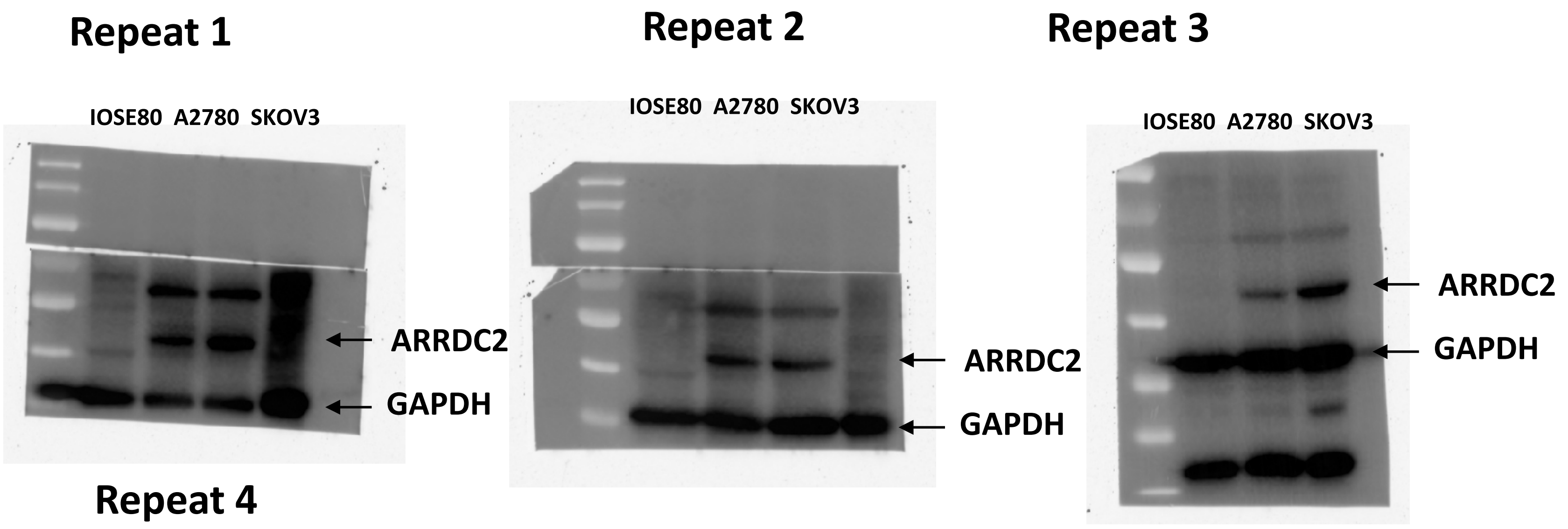

A2780

Example of original western blot

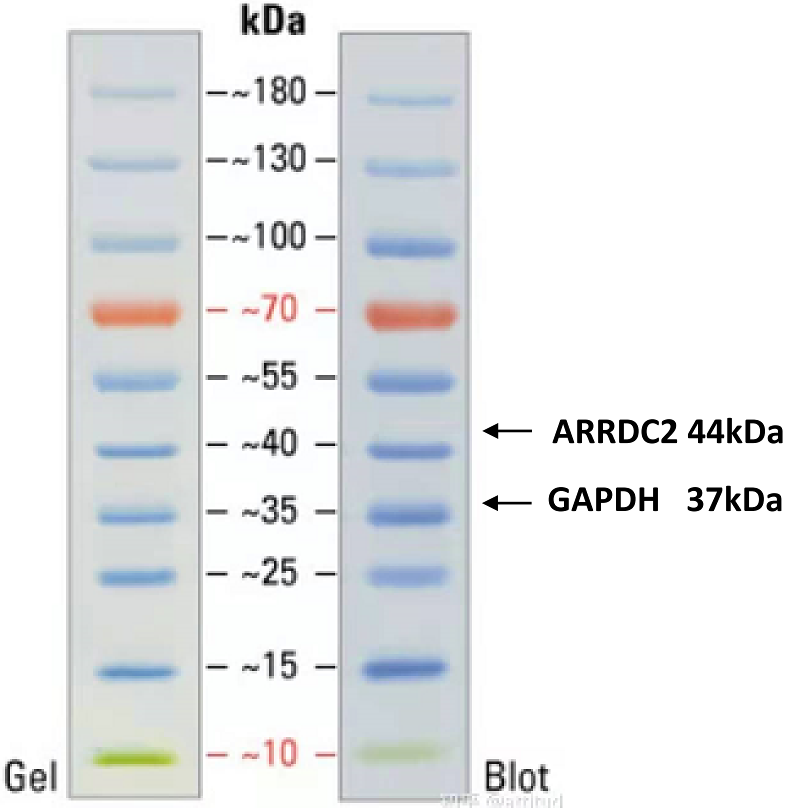

A2780 （Figure 8.D）

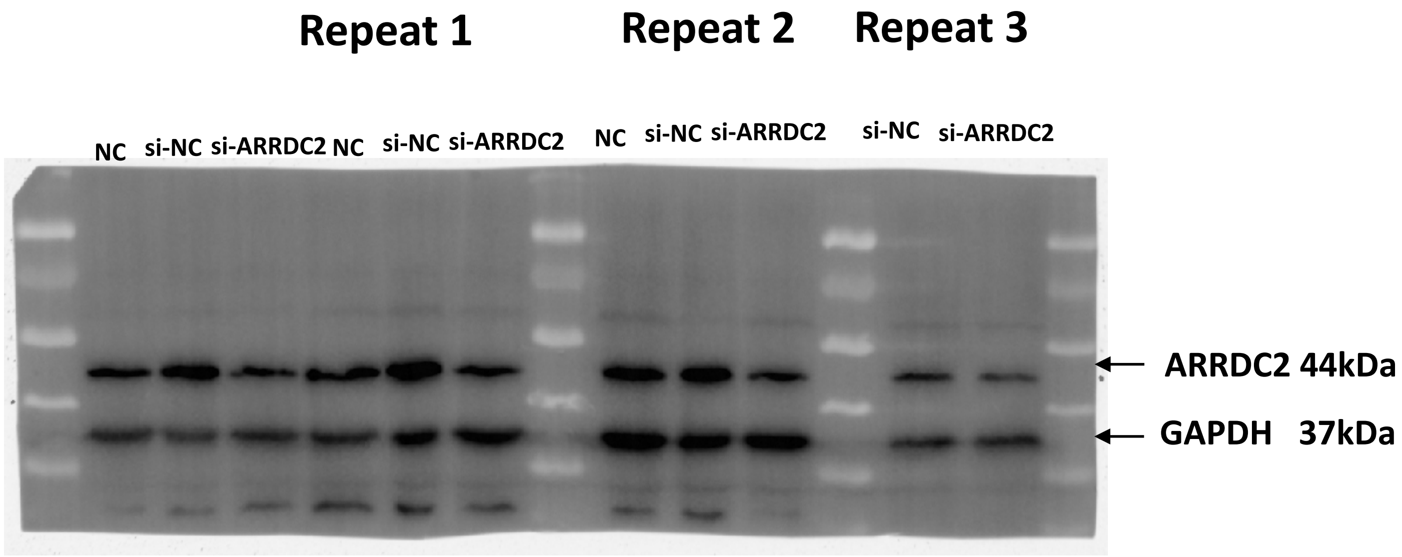

SKOV3

Example of original western blot

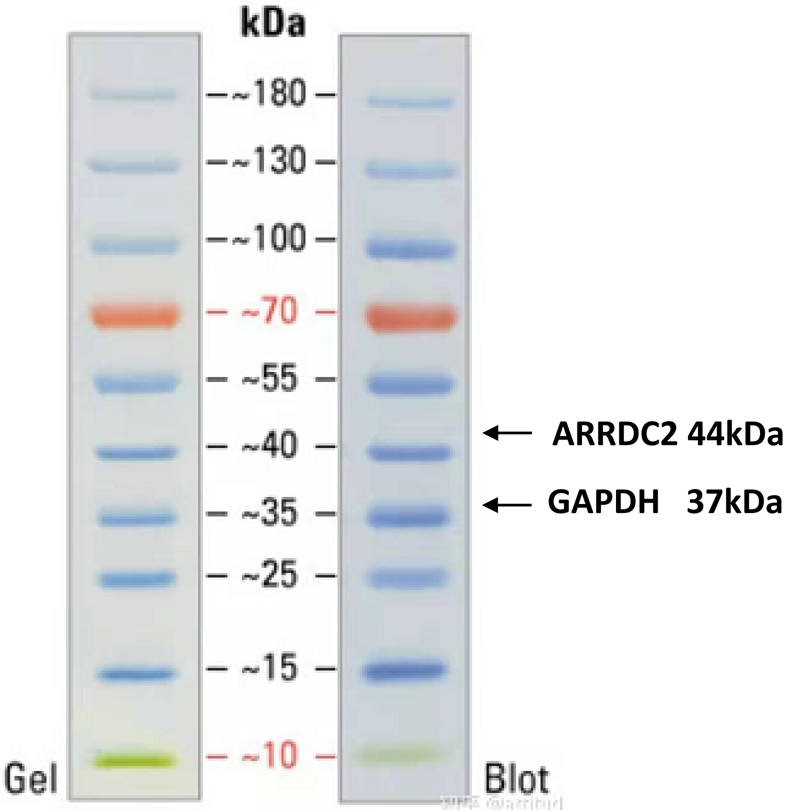

SKOV3 （Figure 8.D）

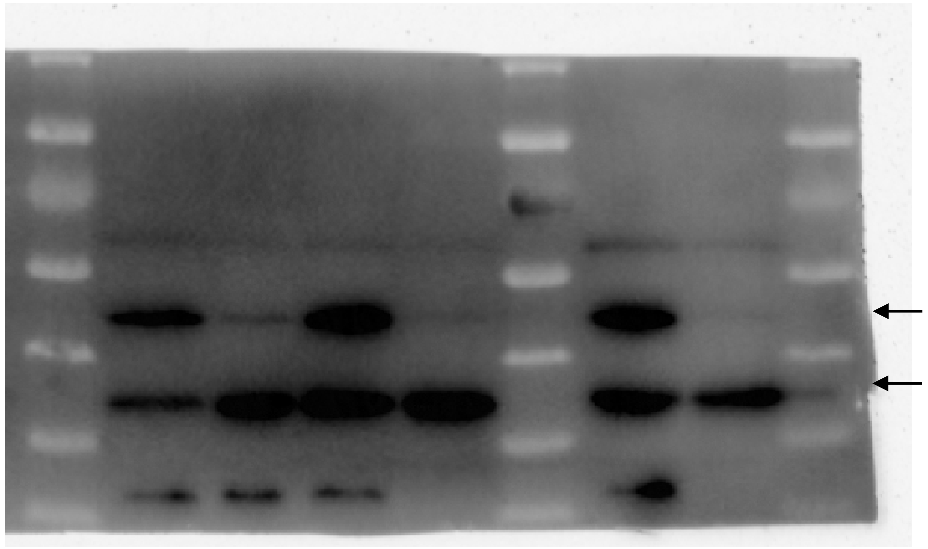

Supplement: Supplementary file 1 [file DataSheet1.PDF]
