# Supplementary material for: A Potential Indicator ARRDC2 Has Feasibility to Evaluate Prognosis and Immune Microenvironment in Ovarian Cancer
Source: Front Genet. 2022 May 18;13:815082. doi: 10.3389/fgene.2022.815082 (PMC9157644; doi:10.3389/fgene.2022.815082)
Supplement: Supplementary file 2 [file DataSheet2.docx]

Supplementary Material


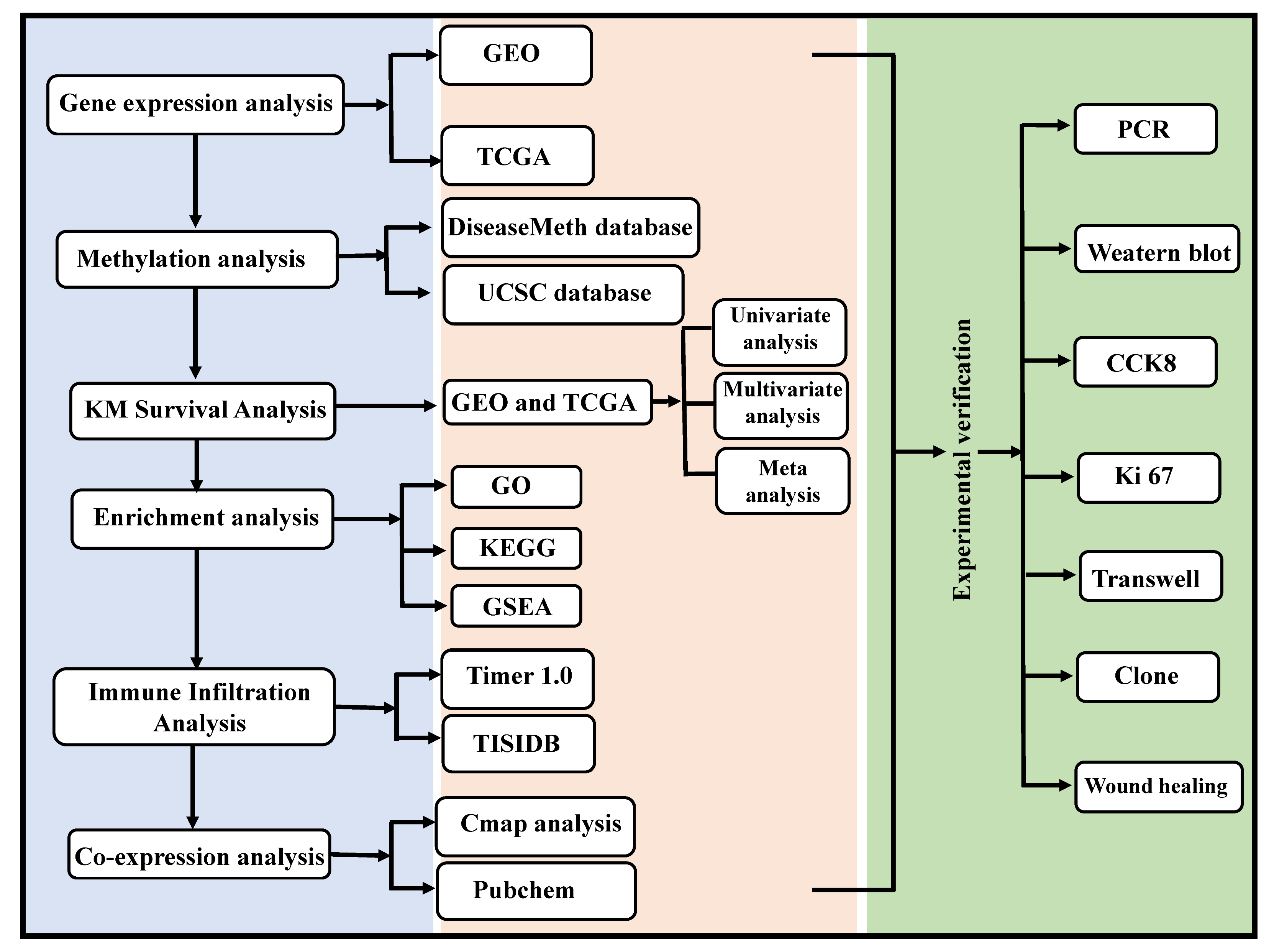


**Figure S1.** Schematic diagram of the flow of this study.


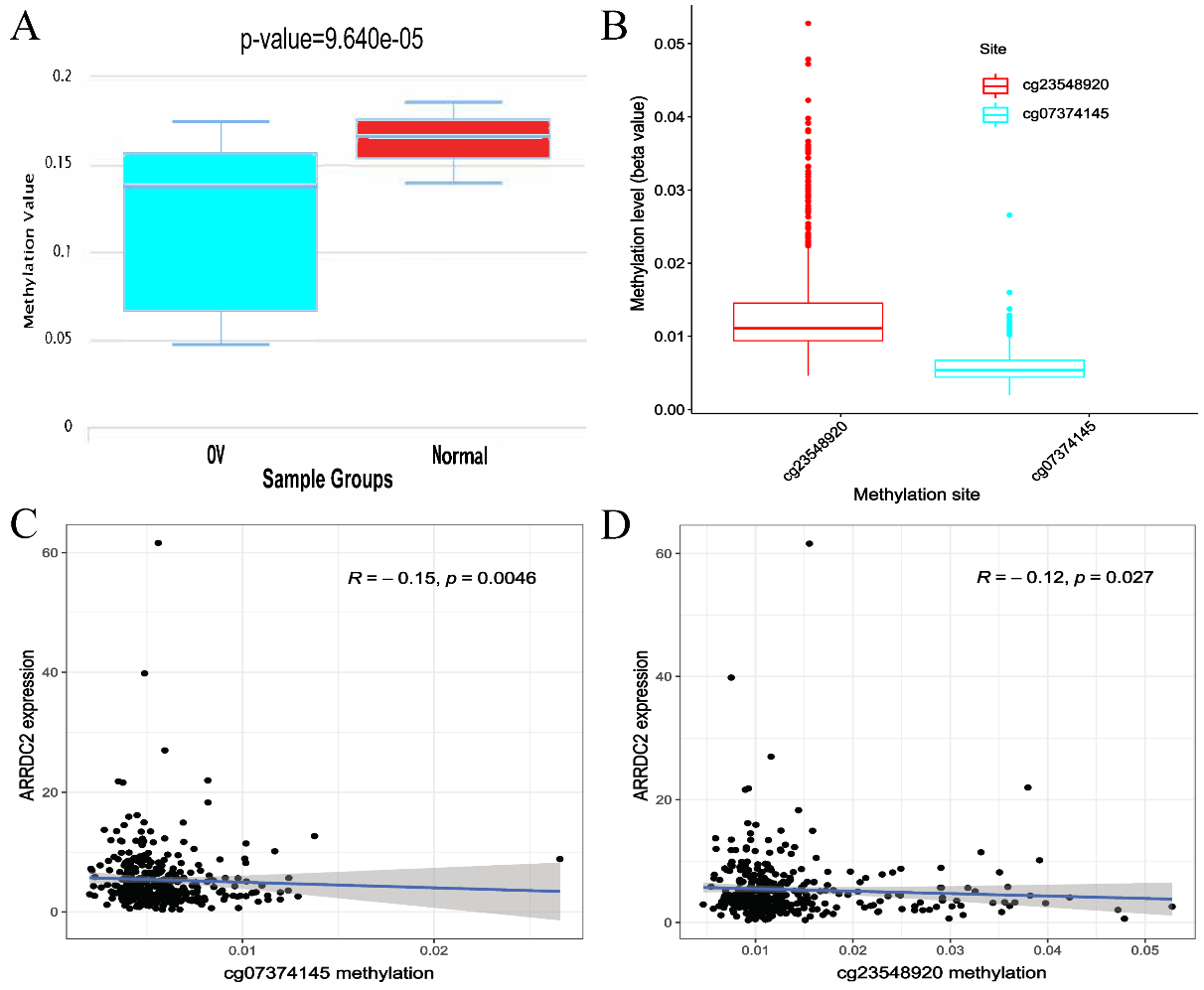


**Figure S2.** Methylation levels of ARRDC2 in ovarian cancer. (Methylation expression and methylation sites) based on the UCSC and The human disease methylation database. **(A)** Box plot of ARRDC2 methylation levels in ovarian cancer from the DiseaseMeth database. The blue color represents the methylation level in ovarian cancer tissues and the red color represents the methylation level in normal ovarian tissues (p-value =9.640e-05). **(B)** Box plot of two hypermethylated sites of ARRDC2 in ovarian cancer, red represents cg23548920 and blue represents cg07374145. **(C)** Correlation of ARRDC2 expression level with the methylation level of cg23548920 locus (R=-0.15, P=0.0046). **(D)** Correlation of ARRDC2 expression level with the methylation level of cg07374145 locus (R=-0.12, P=0.027).

**Table S1. Characteristics of patients with OV based on TCGA**

| Characteristics |  | Number of cases | Percentages (%) |
| --- | --- | --- | --- |
| Age | <=60 | 204 | 54.84 |
|  | >60 | 168 | 45.16 |
| Clinical stage | Stage I/II | 23 | 6.233 |
|  | Stage III/IV | 346 | 93.77 |
| Lymph node involvement | NO | 48 | 12.90 |
|  | YES | 99 | 26.61 |
| Histologic grade | G1 | 1 | 0.279 |
|  | G2 | 42 | 11.29 |
|  | G3 | 318 | 85.48 |
|  | GX | 6 | 1.613 |
| Metastasis | WITHOUT Metastasis | 84 | 22.58 |
|  | WITH Metastasis | 241 | 64.78 |
| Tumor residual disease | No Macroscopic disease | 65 | 17.47 |
|  | 1-10 mm | 170 | 45.70 |
|  | 11-20 mm | 26 | 6.990 |
|  | >20 mm | 69 | 18.55 |
| Venous invasion | NO | 40 | 10.75 |
|  | YES | 62 | 16.67 |
|  | Unknow | 270 | 72.58 |
| Race | Asian | 11 | 2.957 |
|  | Black or African American | 25 | 6.720 |
|  | Unknow | 10 | 2.688 |
|  | White | 326 | 87.63 |
